# Mapping oysters on the Pacific coast of North America: A coast-wide collaboration to inform enhanced conservation

**DOI:** 10.1371/journal.pone.0263998

**Published:** 2022-03-17

**Authors:** Aaron Kornbluth, Bryce D. Perog, Samantha Crippen, Danielle Zacherl, Brandon Quintana, Edwin D. Grosholz, Kerstin Wasson

**Affiliations:** 1 The Pew Charitable Trusts, Washington, D.C., United States of America; 2 Department of Biological Science, California State University Fullerton, Fullerton, California, United States of America; 3 Department of Environmental Sciences, University of California Riverside, Riverside, California, United States of America; 4 Department of Environmental Science and Policy, University of California, Davis, Davis, California, United States of America; 5 Department of Ecology and Evolutionary Biology, University of California, Santa Cruz, Santa Cruz, California, United States of America; 6 Elkhorn Slough National Estuarine Research Reserve, Watsonville, California, United States of America; The University of Sydney, AUSTRALIA

## Abstract

To conserve coastal foundation species, it is essential to understand patterns of distribution and abundance and how they change over time. We synthesized oyster distribution data across the west coast of North America to develop conservation strategies for the native Olympia oyster (*Ostrea lurida*), and to characterize populations of the non-native Pacific oyster (*Magallana gigas*). We designed a user-friendly portal for data entry into ArcGIS Online and collected oyster records from unpublished data submitted by oyster experts and from the published literature. We used the resulting 2,000+ records to examine spatial and temporal patterns and made an interactive web-based map publicly available. Comparing records from pre-2000 vs. post-2000, we found that *O*. *lurida* significantly decreased in abundance and distribution, while *M*. *gigas* increased significantly. Currently the distribution and abundance of the two species are fairly similar, despite one species being endemic to this region since the Pleistocene, and the other a new introduction. We mapped the networks of sites occupied by oysters based on estimates of larval dispersal distance, and found that these networks were larger in Canada, Washington, and southern California than in other regions. We recommend restoration to enhance *O*. *lurida*, particularly within small networks, and to increase abundance where it declined. We also recommend restoring natural biogenic beds on mudflats and sandflats especially in the southern range, where native oysters are currently found most often on riprap and other anthropogenic structures. This project can serve as a model for collaborative mapping projects that inform conservation strategies for imperiled species or habitats.

## Introduction

Foundation species are central drivers of the ecosystems they inhabit [1] and are sometimes referred to as the bedrocks of their local biotic and abiotic communities. Well-known marine and estuarine foundation species include corals, seagrasses, kelps, mangroves, and oysters: where they occur, they are often dominant spatially and in terms of total biomass, and they are responsible for creating the complex habitats upon which many other species rely [2]. They can contribute substantial ecosystem services such as primary production, water filtration, carbon and nitrogen sequestration, sediment retention and shoreline stabilization, and provision of habitat for juvenile fish and invertebrates [3].

Yet numerous marine and estuarine foundation species have declined over time, including shallow water corals [4], seagrasses [5], and mangroves [6], often at least in part due to human influences [7]. This disrupts normal ecosystem functioning, causing declines in associated species that depend on them, which, in turn, can affect the people who live there and derive benefits from the services provided by foundation species. Given foundation species’ outsized roles in producing and maintaining habitat, food, biodiversity, and cultural, economic, and spiritual values, their protection and restoration is key to humans and non-humans [8].

Conserving foundation species requires knowledge about where they currently exist and where they once existed. Such spatiotemporal information allows conservation practitioners to more effectively target their efforts, for example, by protecting the most extensive remaining populations or restoring species to areas that have experienced declines. Thus, *pro-actively* identifying and conserving foundation species is likely the most economical and effective means to sustain their populations and the ecosystem services that they provide [9]. Information on past distribution and abundance usually resides in many different records that can be challenging to integrate. Thorough quantitative characterizations often occur well after declines have begun, either because declines began so long ago and/or because little attention was paid initially to common-place, ubiquitous species. Information on current distribution and abundance can also be challenging to integrate across the large geographic scales that correspond to the ranges of many coastal foundation species.

Oysters are important foundation species that have declined globally [10]. In the Eastern Pacific, the one native oyster species between Alaska, U.S. and Punta Eugenia, Baja California, Mexico is the Olympia oyster, *Ostrea lurida* [11–13]. It declined throughout its range to functional extirpation over several decades spanning the late 1800s to early 1900s before any ecological studies documented the early extent of population sizes or the distribution of oyster beds. The area of their beds in the late 1900s and early 2000s is now estimated at <1% of their former extent [14].

With rare exceptions [15, 16], most prior studies examining *O*. *lurida* distributions were completed at a local or regional scale. For example, there was an early survey for oysters throughout California as a cooperative agreement between the California Division of Fish and Game and U.S. Bureau of Fisheries to investigate the potential for aquaculture [17]. Once the species had already suffered substantial declines, decades passed before additional regional and local surveys were conducted in British Columbia [18] and southern California [19].

**Fig 1:**
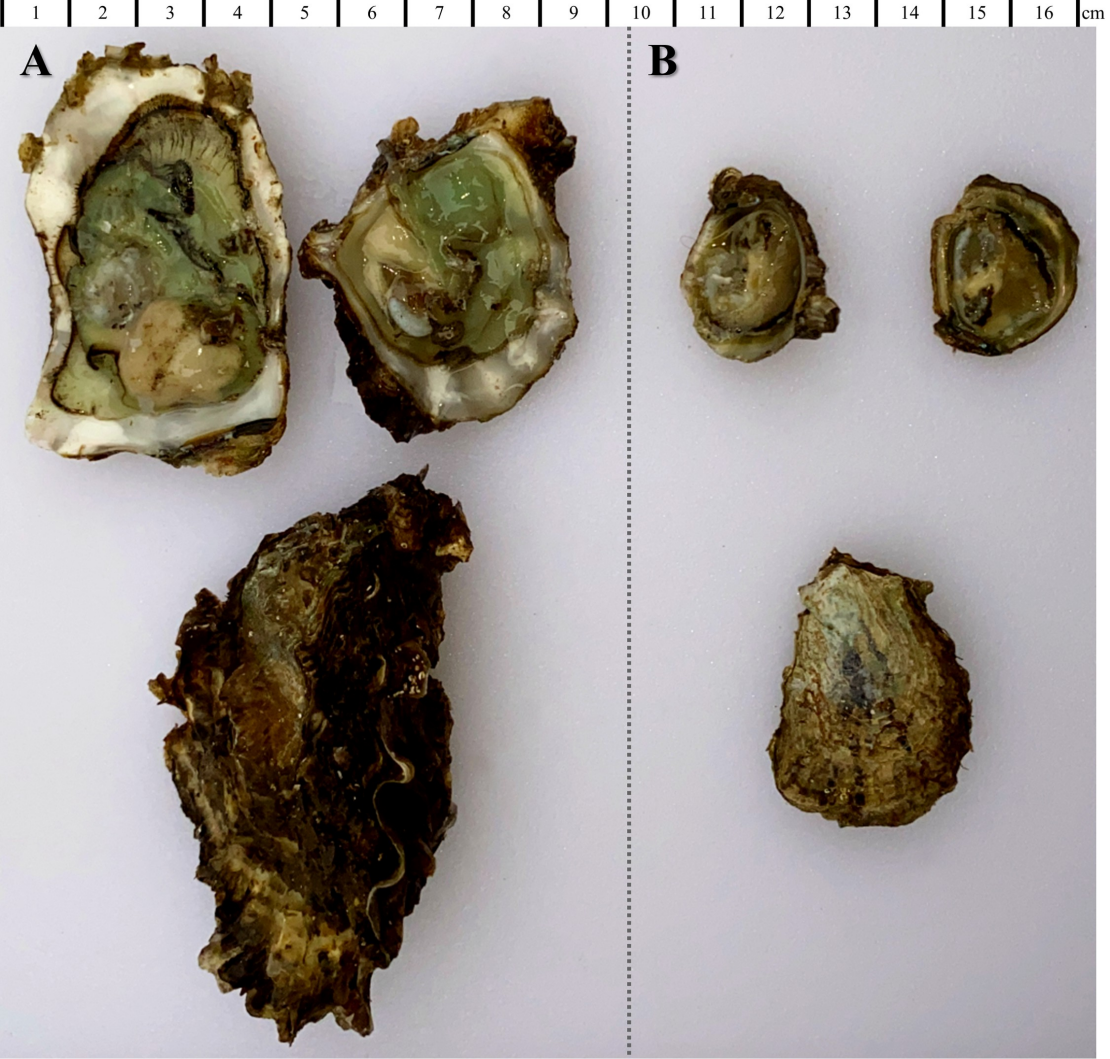
Native and non-native oysters. Photos of *M*. *gigas* (A) and *O*. *lurida* (B) shucked (top row) and intact (bottom row), collected from San Diego Bay, CA in 2021.

The native *O*. *lurida* is no longer the only oyster that is widely established on this coast. *Magallana gigas*, the Pacific oyster, which is native to the Western Pacific, is the dominant aquaculture species in this region. Besides those found in aquaculture farms, wild populations have become established in many estuaries, although its spread has not been well-documented across this coast, excepting Washington [20], and southern California [19, 21]. This species was formerly known as *Crassostrea gigas*, and some debate continues about its nomenclature [22], but we support recent arguments in favor of the use of *M*. *gigas* [23]. Because *M*. *gigas* has spread widely to coasts around the world, with potential for negative impacts on native oyster species [24] and community diversity, structure, and ecosystem processes [25], it is imperative to monitor changes in its distribution and abundance throughout the range of *O*. *lurida*. This underscores the need for an updated range-wide synthesis of the population status of both native *O*. *lurida* and non-native *M*. *gigas*. The species differ in many regards. For example, *M*. *gigas* is larger (Fig 1), grows faster, and free-spawns eggs, while *O*. *lurida* releases larvae after a short brooding period, and typically occurs lower in the intertidal zone [15, 19, 26]. However, they often co-occur within sites and within the mid-intertidal [16, 19] and both perform some of the same ecosystem services, such as water filtration [27].

The primary goal of our investigation was to enhance conservation strategies for *O*. *lurida* along the West Coast of North America by improving understanding of past and present distribution and abundance of the species. This had been identified as a priority by the Native Olympia Oyster Collaborative (NOOC), a collaborative network of oyster scientists, practitioners, educators, and aquaculturists, which synthesized all past restoration efforts for this species up to 2019 [28]. Simultaneously, The Pew Charitable Trusts (Pew) launched an effort in 2019 to map coastal foundation species in the U.S., which proved challenging in the case of oysters on the West Coast due to a lack of synthesized information. Scientists from NOOC and Pew thus joined together to advance priorities of both groups. While our original focus had been on the native oyster on this coast, we also were interested in characterizing the spread of populations of *M*. *gigas* to areas outside of aquaculture. The timing coincided with the first months of the COVID-19 pandemic, which provided an opportunity to solicit unpublished oyster records from researchers who were working from home rather than conducting fieldwork. We established protocols for interacting remotely that allowed us to capitalize rapidly on the unprecedented availability of oyster researchers, gathering their records of the native and non-native oyster into a single coast-wide database. Interns, working virtually, entered previously published records, resulting in the most comprehensive database to date of oyster distribution and abundance on this coast.

The novel database we created allowed us to identify temporal and spatial changes in *O*. *lurida* and *M*. *gigas* distributions and abundances, in particular comparing the “current” period (2000–2020) to earlier times, and determining how their distribution and abundance differ. We characterized spatial patterns to determine whether there were differences across regions, which was only possible due to the consistent methodology we applied range-wide. We conducted spatial analyses of adult distribution to characterize how sites might be interconnected through larval dispersal. We also quantified the substrates on which oysters occurred, as well as how these substrates varied among regions and species. Our main objective was to develop concrete recommendations for improved oyster restoration on this coast. Another objective was to provide a compilation of past and current oyster records for multiple scientific and conservation purposes in the future, facilitated by a user-friendly, interactive online map. A final objective was to create a model for collaborative and interactive mapping projects for other foundation species.

## Methods

### Overview of mapping team and process

Because it may be useful for future mapping efforts by others, we describe here the coordination and staffing for this project. A small team from NOOC and Pew coordinated the effort. We determined which data would be readily feasible to collect (including from historical sources and from experts providing personal records) and designed an interactive data collection tool using ArcGIS Online® by Esri®. We solicited feedback from about a dozen NOOC members on the draft data entry protocols and adapted the protocols based on responses. We then invited NOOC experts from British Columbia to Baja California to participate and provided training on how to enter data correctly into the Portal. Collaborators included academics and staff from federal, state, and local resource management agencies and non-governmental organizations, all of whom possess extensive knowledge about *O*. *lurida*. The community of scientists and practitioners working on *O*. *lurida* along this coast is relatively small and closely affiliated, so we are confident that we included everyone who may have robust records for multiple locations. Three trained student interns on our team entered records from peer-reviewed and grey literature into the database and supported NOOC members in entering their own data. Our coordinating team conducted quality control of the records as well as analyses and syntheses. This project was launched in April 2020; data collection concluded by July 2020. Thus, data collection was completed rapidly, with relatively light personnel investment but a high degree of participation from a network of experts.

### Data collected

We limited data collection to a few components considered most critical for understanding the distribution of oysters and for conservation implications. For each record, there were seven required and six optional elements (Table A in S1 File). The data entry form was short and allowed for quick entry of data (<5 min. per new record). Furthermore, much of the published literature contained only limited ancillary information on oyster occurrences, so it seemed reasonable to focus on key elements of those data, too. A small number of participating oyster experts have much more extensive monitoring datasets available (*e*.*g*., oyster densities from quadrats along permanent transects visited at multiple sites and over multiple years), but we chose not to include this level of detail since it was available for few places and methodologies differed greatly among regions.

The unit of data collection was the “site,” defined as a point at the center of a 20-m stretch of low intertidal shoreline: 10-m on either side of the coordinates entered (or a 20-m diameter circle surrounding a subtidal point, though few subtidal records were provided). To create a straightforward index of abundance, we asked collaborators to estimate if living oysters were “common” (>100), “rare” (1–100), merely “present” at the site, or “absent.” At an intertidal site with a steep slope, the zone occupied by oysters and visible from shore (from about Mean Sea Level to Mean Lower Water) spans only a few meters horizontally, but at a site with a very gentle slope, this can be 100-m wide. Thus, the area of intertidal in a 20-m stretch of coast varies by site and the abundance assessment cannot readily be translated to density per square meter.

We attempted to obtain and enter all readily available oyster records from the entire range of *O*. *lurida*. While this is by far the most extensive compilation of *O*. *lurida* records to date, it certainly is not comprehensive; there are doubtless unpublished monitoring datasets, gray literature reports, and published records embedded in larger datasets that were not included because we did not find them or have access to them. The on-line database we created can be updated in the future as more records are located. For *M*. *gigas*, we attempted to obtain all records within the *O*. *lurida* range and to the north of it so that we could identify the northern range limit on this coast. On the southern end, we stopped searches and data entry at the southern range limit of *O*. *lurida*, recognizing that aquaculture and possibly wild populations of *M*. *gigas* extend considerably farther south.

### Data entry options

Multiple data entry methods were provided to collaborators, including the Portal, which was designed to gather records one-by one, and a Microsoft Excel sheet formatted to collect the same data as the Portal, but in batches and only when precise record coordinates were known. We created and shared a Portal user’s guide as well (S2 File). The Portal was designed to minimize extraneous content but provided essential reference layers (summarized in Table B in S1 File) to help experts identify precise locations to place oyster records. Users were able to place oyster records as points and move them as needed in our editable, hosted feature point layer configured with a custom HTML pop-up. We distributed the Portal to collaborators by invitation only, tracked entries and updates by user, and offered query and data export capabilities. The Portal used the basemap “imagery with labels” because it provides the highest resolution compared to other built-in ArcGIS Online basemaps, allowing users to zoom in to pinpoint the location(s) of their record(s). Portal setup proved relatively straightforward, user feedback was generally positive, and there was a short learning curve, indicating that this interactive, online data-gathering approach is one that may have broad appeal.

### Data sources

Data entered into the Portal were collated from three major data sources: 1) records entered by local experts; 2) published records entered by interns; and, 3) iNaturalist records verified and entered by interns. Published records were gathered from peer-reviewed and grey literature from the past 250 years found in a comprehensive database of shared oyster literature compiled by oyster experts and through Google Scholar. We excluded archeological or paleontological records from our study because we felt these older records would not be relevant for current conservation and management issues, but another effort could synthesize these records to better understand the baseline abundance of *O*. *lurida*. We recognize that *O*. *lurida* has been present for hundreds of millennia [29] and Indigenous peoples have long harvested this species [30]. Much of the historic data we examined did not include exact locations, so they were assigned locations as close as possible to the described portions of the estuaries in which they were documented.

We also mined peer-reviewed and grey literature using Google Scholar searches for keywords “oyster,” “*Olympia oyster*,” “*Ostrea lurida*,*”* “*Ostrea conchaphila*,*”* (a name applied to *O*. *lurida* for some decades), “Pacific oyster,” “*Magallana gigas*,” and “*Crassostrea gigas*.” When we were unsure about how to correctly site and/or interpret oyster records gleaned from these searches, we contacted regional experts for more information.

We also collected data from iNaturalist using the keywords “*Ostrea lurida*” and “*Magallana/Crassostrea gigas*” from British Columbia to San Diego, California. Records from Baja Mexico were not included because there are multiple oyster species that are found in this region [13] and we had a lower confidence in accurate identification from photographs. A trained student intern reviewed all iNaturalist entries and only accepted entries if accurate oyster identification was possible and the oyster could be determined as having been alive at the time of the observation. Identification based upon iNaturalist entries relied upon use of external morphological characters that could be observed in photographs. *M*. *gigas’* external shell has foliations (which often get worn away with age), a thick profile, and reaches a larger maximum size than *O*. *lurida*, while *O*. *lurida’s* external shell lacks significant foliations and is smooth and thin [12]. We excluded iNaturalist records that contained only dead oysters (identified by gaping shell), records that were reported as being on land or in deep water, had geoprivacy enabled (resulting in distorted coordinates), or had low positional accuracy (>20-m radius). The substrate on which the observed oysters were documented was inferred from observer notes, associated iNaturalist photos, and Google Earth (especially seawalls and riprap). Oysters were deemed “common” if it could be inferred based on scale of the photo and if 100+ oysters per 20 m^2^ could be extrapolated, but were otherwise recorded as “present but unknown abundance.”

### Spatial analyses

Upon completing the collection of oyster records via the Portal and desktop research, we analyzed the data spatially using Esri ArcMap™ 10.7 and ArcGIS Pro® 2.7. All data were projected into the North America Albers Equal Area Conic projection as it is suited for equal-area mapping in mid-latitudes. Data provided by Fisheries and Oceans Canada and several collaborators in the U.S. and Mexico were considered “sensitive,” meaning that there may be negative consequences, such as poaching, if the exact coordinates or detailed location descriptions were shared publicly. Spatial analyses for this paper were performed using precise coordinates of these sensitive records as they were provided to us. For the purposes of sharing these data publicly, however, we removed the coordinates of those records from our database and removed the records entirely from the Portal (though they were used to generate the networks described below).

#### Larval and adult networks

**Fig 2:**
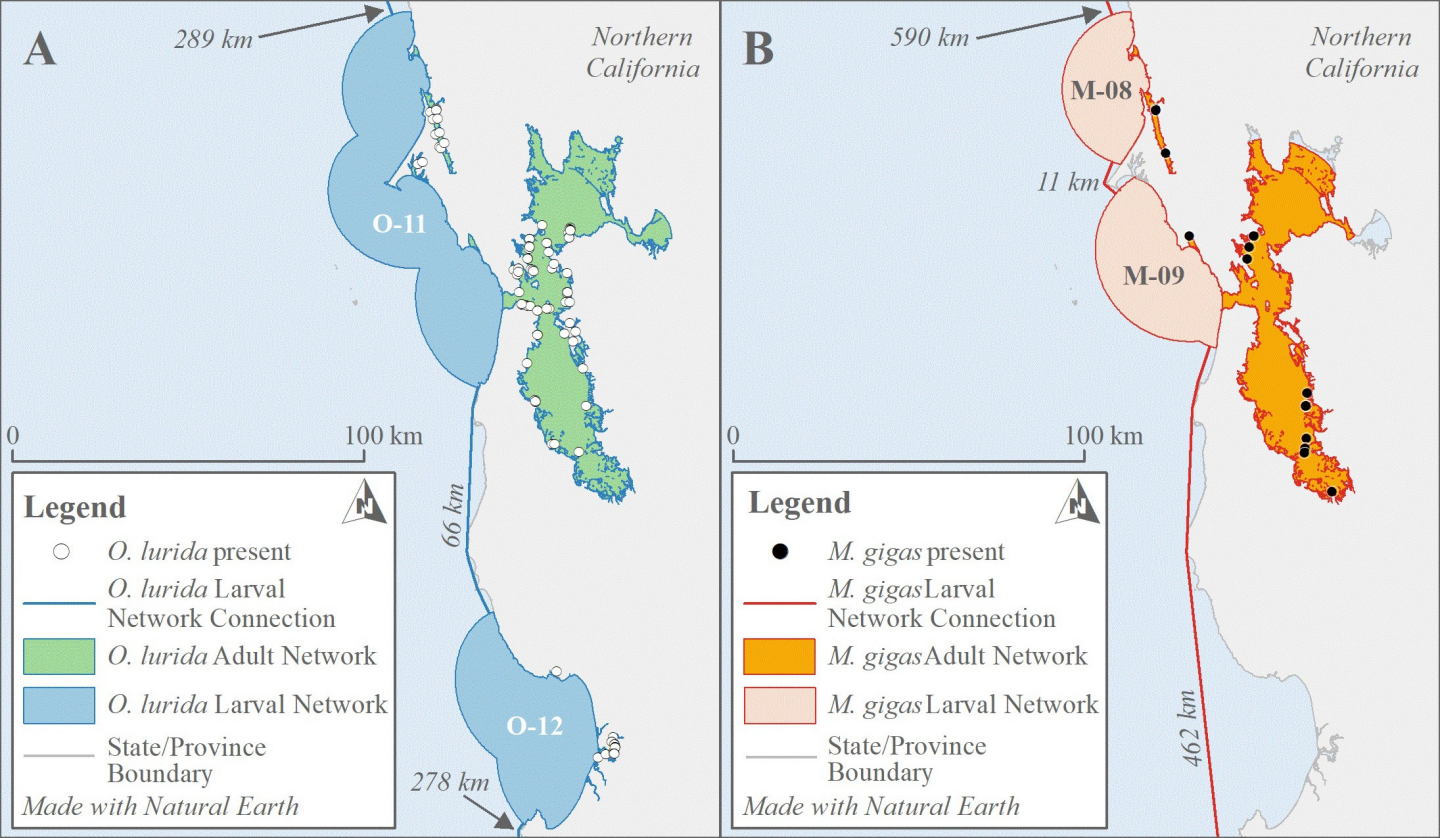
Oyster distribution networks. Examples of oyster larval and adult networks and larval optimal network connections from the San Francisco Bay area (Northern California) for *O*. *lurida* (A) and *M*. *gigas* (B). For *O*. *lurida*, the distance between the San Francisco Bay larval network (O-11) and the next closest network to the south (O-12) is due to oysters present in the Monterey Bay area, which is a shorter distance than for *M*. *gigas*, for which the next closest adults to the south (contained within network M-10, not pictured) are in Southern California.

We generated “networks” that encompassed groups of sites with adult oysters present, which might plausibly be linked through larval dispersal (see Fig 2 for example). *O*. *lurida* larvae can successfully recruit after traveling as far as 75 km if currents are favorable [31], but 30 km was used as a limit to dispersal by Wasson *et al*. [32]. They demonstrated that the network size defined by this metric correlated significantly with stability in recruitment (*i*.*e*., larger networks had less recruitment failure). For both species, we thus considered sites with living adult oysters observed between 2000–2020 and within 30 km of each other to be part of the same *larval network*.

To create larval network polygons, we began by using the data listed in Table C in S1 File to develop a merged, medium-high resolution vector polygon layer representing much of the North American west coast coastline, including estuaries and islands, from Alaska, U.S. to Baja California, Mexico, since there was no readily available dataset at such resolution that spanned this area. We created a separate, merged layer of estuaries (Washington, Oregon, and California, U.S. and Baja California, Mexico) and physical ecosections (British Columbia, Canada) also using data in Table C in S1 File. We performed basic spatial analyses (*e*.*g*., size, centroid) on estuaries and ecosections. We performed a Spatial Join of all oyster records to country, state/province, and estuary/ecosection. The analysis was conducted separately for each oyster species.

To enhance processing speed, we converted the merged land/coastline vector layer to a raster layer (cell size = 10 m) and then used the Dice tool on the resulting raster with a limit of 10,000 vertices. For oyster records that intersected the diced raster, we used the Snap tool to move them to the nearest edge of the raster so that they would not be excluded from the next analysis step. The maximum distance that we snapped records to the raster layer’s edge was 1 km; this was done so that we could include records whose positional accuracy may have been low. Using only oyster records marked present, rare, and/or common and observed within 2000–2020, for each species, we created a 10-m buffer around each record and converted the resulting vector polygon layer to a 10-m resolution raster layer. To create the larval networks, we input the previously generated raster layer into the Distance Accumulation tool, which describes each feature’s relationship to a source or a set of sources based on the straight-line distance while accounting for barriers (in our case, the coastline), where maximum distance = 30 km based on [32] and cell size = 10 m. Parameterized as described here, this tool effectively generated a 30-km buffer around each oyster record following the shortest straight-line distance while also accounting for barriers (that is, not simply “as the crow flies”). Where there are overlapping 30-km buffers from three or more points, those buffers are merged into a single polygon that represents one larval network.

Some of the area within larval networks was located in open ocean (*e*.*g*., not within an estuary and/or in very deep water) where oysters are not likely to live, so we defined *adult networks* as the subset of any larval network only within well-delineated estuaries (*i*.*e*., not in the open ocean). To create the adult networks, we used the Clip tool to select only the portion of larval networks that overlapped the merged layer of estuaries described above. Since we did not have high-resolution estuary boundaries for British Columbia, Canada, we only developed larval, and not adult, networks there.

#### Larval network connectivity

To determine connectivity among larval networks (separately for *O*. *lurida* and *M*. *gigas*), we first ran the Con tool on the larval networks as an intermediate step where true constant value = 1. We used the Optimal Region Connections tool where cell size = 10 m, input features = larval networks, input barriers = diced coastline/land raster polygon, and with “no connections” set for connections within regions. The output of this analysis measures the shortest, *i*.*e*., optimal, distances among networks while accounting for barriers. For each network, we calculated the mean distance to the nearest network to the north and the nearest network to the south as the metric for network isolation (*e*.*g*., higher numbers represent more isolated networks).

### Statistical analyses

#### Geographic units

We used multiple spatial categories for our comparative analyses and syntheses. At the largest scale, we compared the Northern, Central, and Southern portions of the distribution. At a somewhat smaller scale, we compared states/provinces. Note that for simplicity, we refer to British Columbia, Canada as “Canada” and Baja California Norte and Sur, Mexico as “Mexico” in some figures and tables, and that the largest U.S. state, California, was divided into northern and southern portions at Point Conception, a widely recognized biogeographic boundary [33]. At a finer scale, we compared larval networks (described above). Finally, at the smallest scale, we evaluated oyster distribution and abundance among individual estuaries. For much of the North America west coast, estuaries are small and easily distinguished, so categorizing records into estuaries is straightforward. North and east of Washington’s Cape Flattery, oyster populations are more continuous without clear boundaries. For these areas, we used the Washington Department of Fish and Wildlife’s Puget Sound basins [34] (*e*.*g*., Hood Canal, Whidbey Basin) and British Columbia DataBC’s marine ecosections [35] (*e*.*g*., North Coast Fjords, Hecate Strait) to place records into separate “estuaries.” Below, we refer to this level of analysis as being by estuary even though they are not strictly estuaries.

#### Distribution index

To evaluate how broadly distributed each oyster species was, we divided the number of records with the species present in the estuary by the total number of records (presence and absence) for the species in the estuary. A score of 0 signifies that all records for this estuary were of absence; a score of 1 indicates that all records for this species in this estuary were of it being present (no absence records). This index is sensitive to search effort and reporting of negative results (absence of oysters from sites), which typically increased over time. Early records often simply noted a few sites with presence of a species, while later investigations involved thorough searches and reporting of sites with absences as well as presence records.

#### Abundance index

To evaluate how abundant each oyster species was in estuaries or regions where it is present, we divided the number of “common” records for each species by the total records for the estuary that provide abundance information (“common” plus “rare” abundance records). A score of 0 signifies that all records that provided abundance data were “rare” records; a score of 1 indicates that all records that provided abundance data were “common” records. The Abundance Index should be less sensitive to changes in search effort and reporting of negative results than the Distribution Index because it does not include absence records. We examined the relationship between the two indices by plotting them against each other for each species (Fig A in S1 File) and calculating the Kendall rank correlation. The two indices were not correlated for *O*. *lurida* (R = 0.36, p = 0.79), suggesting that they are assessing different components of this species’ ecology and are worth calculating and reporting separately. However, they were highly correlated for *M*. *gigas* (R = 0.59, p < 0.0001).

#### Change in distribution and abundance indices

To evaluate temporal changes, we compared pre-2000 to post-2000 indices because there was a natural break in the number of records and because this millennial break-point is a conventional temporal boundary to consider. We calculated Distribution and Abundance Indices for each estuary, and compared these pre-2000 vs. post-2000 using a paired Wilcoxon test (each estuary was a replicate for comparing the index in the two time periods). We only included estuaries that had at least three records in both periods. For *O*. *lurida*, this yielded 21 replicates (estuaries) for the Distribution Index and 17 for the Abundance Index. For *M*. *gigas*, there were 10 replicates for the Distribution Index. Since only a single estuary (Vancouver Island shelf area) had both pre-2000 and post-2000 *M*. *gigas* abundance information, no analysis of change in abundance over time was carried out for this species.

#### Broad regional patterns among networks

To quantify differences among larval networks among broad regions, defined here as North (Canada and Washington), Central (Oregon and Northern California) and South (Southern California and Mexico), we drafted box plots and conducted a Kruskal-Wallis test for network size and for isolation of each species. We used the larval network as replicate for this analysis, which involved fairly limited sample sizes. For *O*. *lurida*, there were 6, 6, and 2 networks for the North, Central, and South, respectively; for *M*. *gigas*, 6, 3, and 3, respectively.

#### Regional differences in indices

To evaluate differences among Canada, Washington, Oregon, Northern California, Southern California, and Mexico, we drafted box plots and conducted a Kruskal-Wallis test for the Distribution and Abundance Index of each species, using data from post-2000. We used estuary as replicate for this analysis. We also plotted indices vs. latitude using estuary as replicate. As the relationship was distinctly non-linear, we used local polynomial regression fitting for visualization. We plotted indices vs. estuary area with linear regressions and calculated the Kendall rank correlation.

#### Comparison of indices and networks between species

To evaluate differences in the indices between *O*. *lurida* and *M*. *gigas* post-2000, we calculated the average Distribution and Abundance Index per estuary and compared these between species using a paired Wilcoxon test with statistics and graphing as described above. We used only estuaries that had at least three records for each species, which yielded 39 for the Distribution Index and 19 for the Abundance Index. To evaluate differences in size and isolation of networks, we also used a paired Wilcoxon test with networks in the same areas compared to each other. To examine relationships among the indices within and between species, we plotted them against each other and calculated the Pearson correlation coefficient.

#### Substrate analysis

We analyzed the substrate types on which *O*. *lurida* and *M*. *gigas* were observed between 2000–2020 and how this differed by region. Substrate classifications were based, in part, on approximate mean diameter, and included: 1) riprap or boulder (>25 cm); 2) cobble (5–25 cm); 3) gravel/pebble (0.6–5 cm); 4) sandflat/mudflat; 5) seawall/dock/piling; and, 6) other anthropogenic structure. To determine whether substrate use differed geographically, we conducted a chi-square test comparing percentage of each substrate type by region for each species separately. We eliminated regions from the analysis if they had fewer than 5 observations in any category (*e*.*g*., Mexico for both species, and additionally Oregon and Northern California for *M*. *gigas*). We also conducted a chi-square test comparing substrate use by species to directly compare whether the native and non-native species were recorded in different proportions on various habitats. For this comparison, we consolidated all records across all regions.

To explore whether regional differences in substrate use were driven by differences in substrate availability, we quantified the availability of different substrates vs. their use by oysters in two estuaries, San Diego Bay, California and Puget Sound, Washington, using the same six substrate types as above. For San Diego Bay, substrate availability was determined by calculating the area of each substrate type by first totaling the perimeter of each habitat type throughout the bay using the measurement tool on Google Earth (as length) and estimating the width of each habitat type using field-calibrated measures. For Puget Sound, substrate availability by type was extracted from Washington’s Shoreline Habitat Classifications [36] and then reclassified to match the six substrate types above. To determine if substrate use by oysters in the two estuaries was determined by habitat availability versus habitat preference, we ran Fisher’s Exact Tests (versus Chi-Squared analyses, to account for categories that had fewer than five records) and calculated a selection index (*w*_*i*_ = *u*_*i*_*/p*_*i*_) where values >1 indicate that the habitat is used proportionally more than available and < 1 indicate that the habitat is used proportionally less than available. To understand if use was actually significantly different than availability, we calculated the 95% confidence intervals around the proportions of substrates used. If the habitat availability value (*p*_*i*_) value fell outside of the 95% confidence interval for use (*u*_*i*_), we concluded that the habitat was recruited to significantly more or less than its availability, indicating preference or avoidance [37].

Our characterization of habitat use by both oyster species carries some important caveats. Firstly, these characterizations rely upon observations by people who may be “sampling” the habitats relative to their availability, especially for iNaturalist records, and may be biased toward habitats most easily accessible or most often used for research and restoration. Next, both the calculation of shoreline habitat by our group in San Diego Bay and the characterization of habitat availability in Washington’s Shoreline Habitat Classifications did not include explicit reference to particular tidal elevations available for use by *O*. *lurida* and *M*. *gigas*, which are both known to associate with particular tidal elevations [19]. Hence, our comparisons of habitat usage across locations and relative to availability should be considered a first broad-brush attempt.

#### Statistical analyses

The substrate analyses were conducted using JMP® 14.1.0. All other analyses described above were conducted using R version 3.6.2 [38]; associated graphs were made with the ggplot [39] and ggpubr [40] packages. The Distribution and Abundance Indices were not normally distributed due to many values of 0 and 1, leading to the choice of non-parametric tests as detailed above.

## Results

**Fig 3:**
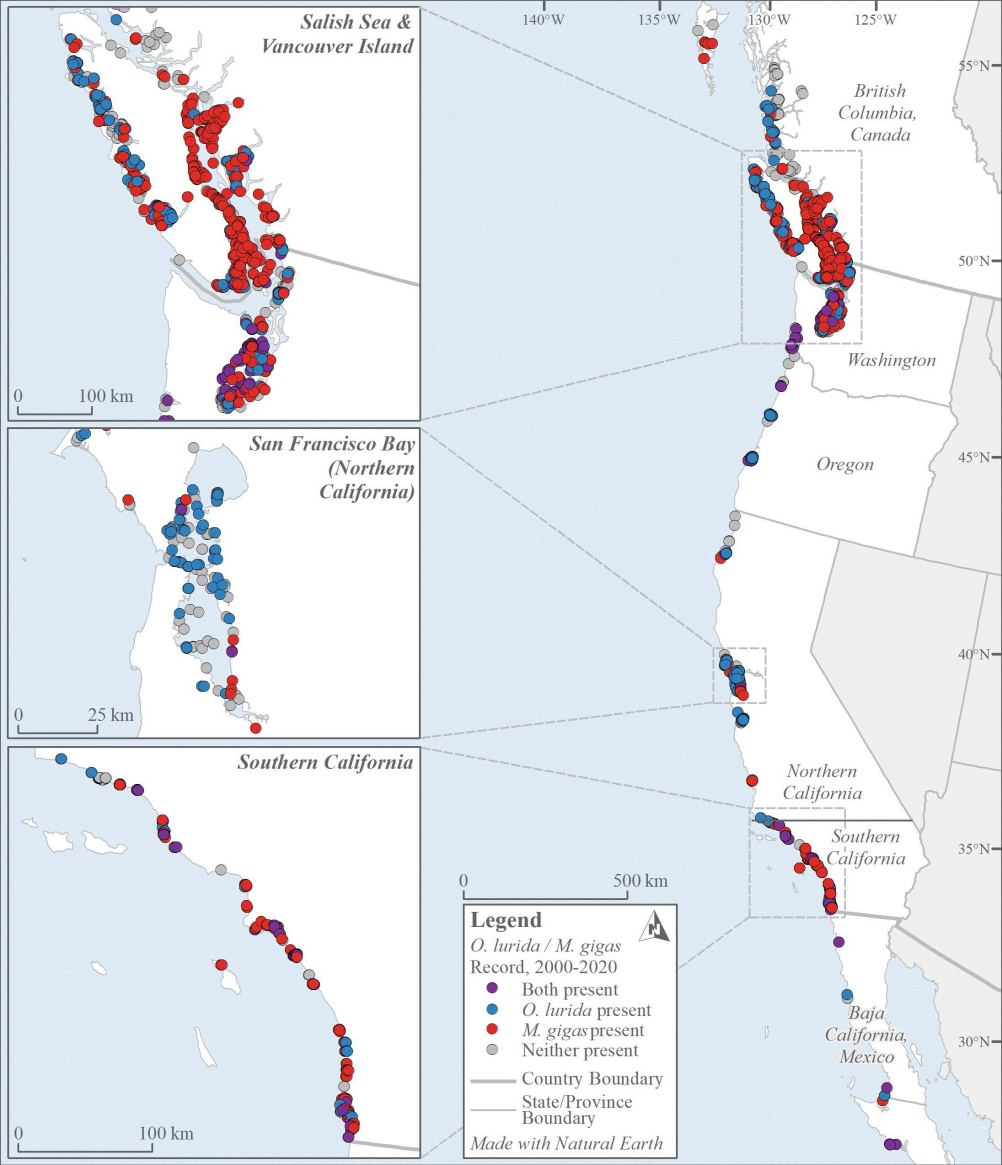
North American west coast coast-wide distribution of *O*. *lurida* and *M*. *gigas*, 2000–2020. Records are color-coded according to whether one, the other, both, or neither species were documented as present during this period. Note that in some cases, records overlay each other. An interactive map of these data is available (S4 File).

**Fig 4:**
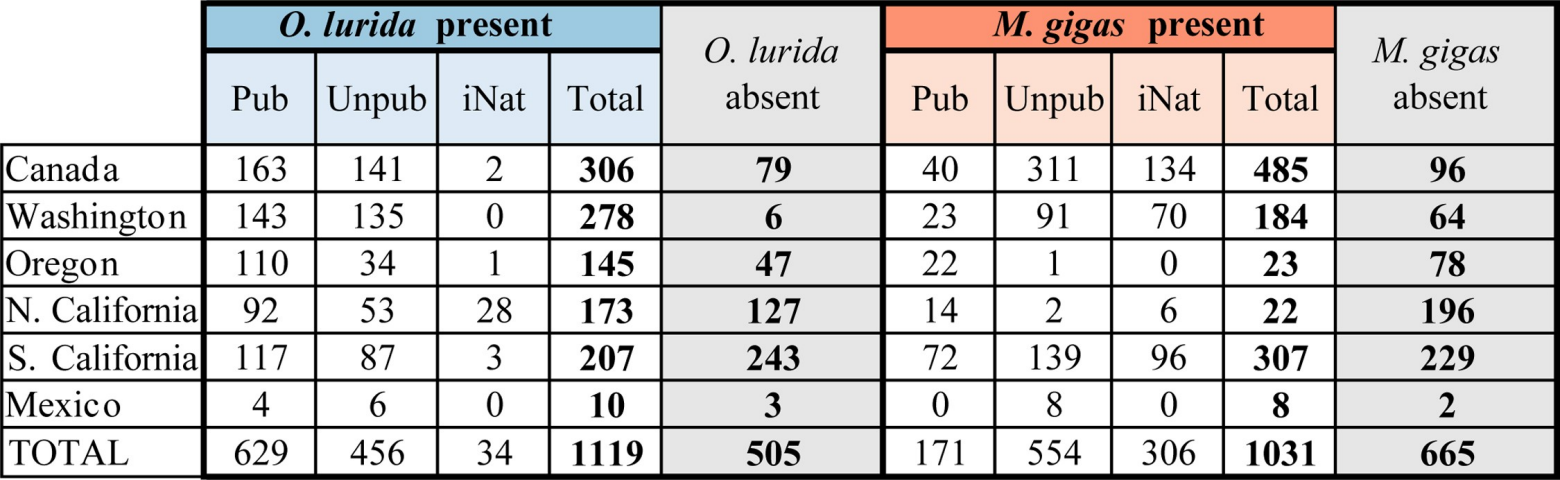
Summary of oyster records. This table shows the number of records by presence/absence, region, and publication type for *O*. *lurida* and *M*. *gigas*. Grey columns indicate total records by region. Note that individual records sometimes contain data for both species, meaning the total sums of records by species exceed the total number of records collected. Abbreviations: Pub = Published, Unpub = Unpublished, iNat = iNaturalist.

We collected 2,296 oyster records made by over 150 observers from 1602 to 2020; these data are archived and publicly available (S3 and S4 Files). These records spanned many thousands of kilometers of coastline and 34.25 degrees of latitude (Fig 3). The data include over 1,600 records each for *O*. *lurida* and *M*. *gigas*. Both species had more records of presence (denoted in the database as either present, rare, or common) than absence: 69% of *O*. *lurida* vs. 61% of *M*. *gigas* records (Fig 4). Most records were located in estuaries (only 10 records were from open coast areas, *i*.*e*., in-between well-defined estuary boundaries).

The sources of data varied by species. Most *O*. *lurida* records of presence originated from publications (56%) versus unpublished (41%) and iNaturalist records (3%). Most *M*. *gigas* records of presence originated from unpublished records (54%), compared to iNaturalist (30%) and published literature (17%). *M*. *gigas* was recorded on iNaturalist an order of magnitude more often than *O*. *lurida*. Canada, Washington, and Southern California comprised 71% of *O*. *lurida* and 95% of *M*. *gigas* presence records, whereas Mexico comprised just 1% of total records for both species (Fig 4). We suspect some of these geographic and species differences are due to frequency of human encounters rather than actual differences in oyster abundance (e.g., *M*. *gigas* is bigger and occurs higher in intertidal, so is more readily spotted at public access locations like harbors than *O*. *lurida*; frequency of observations around big cities like Seattle or Los Angeles reflects high human population densities while the dearth of observations in Baja California reflects low human densities there).

### Temporal patterns

#### Change over time in state of knowledge about oysters on this coast

**Fig 5:**
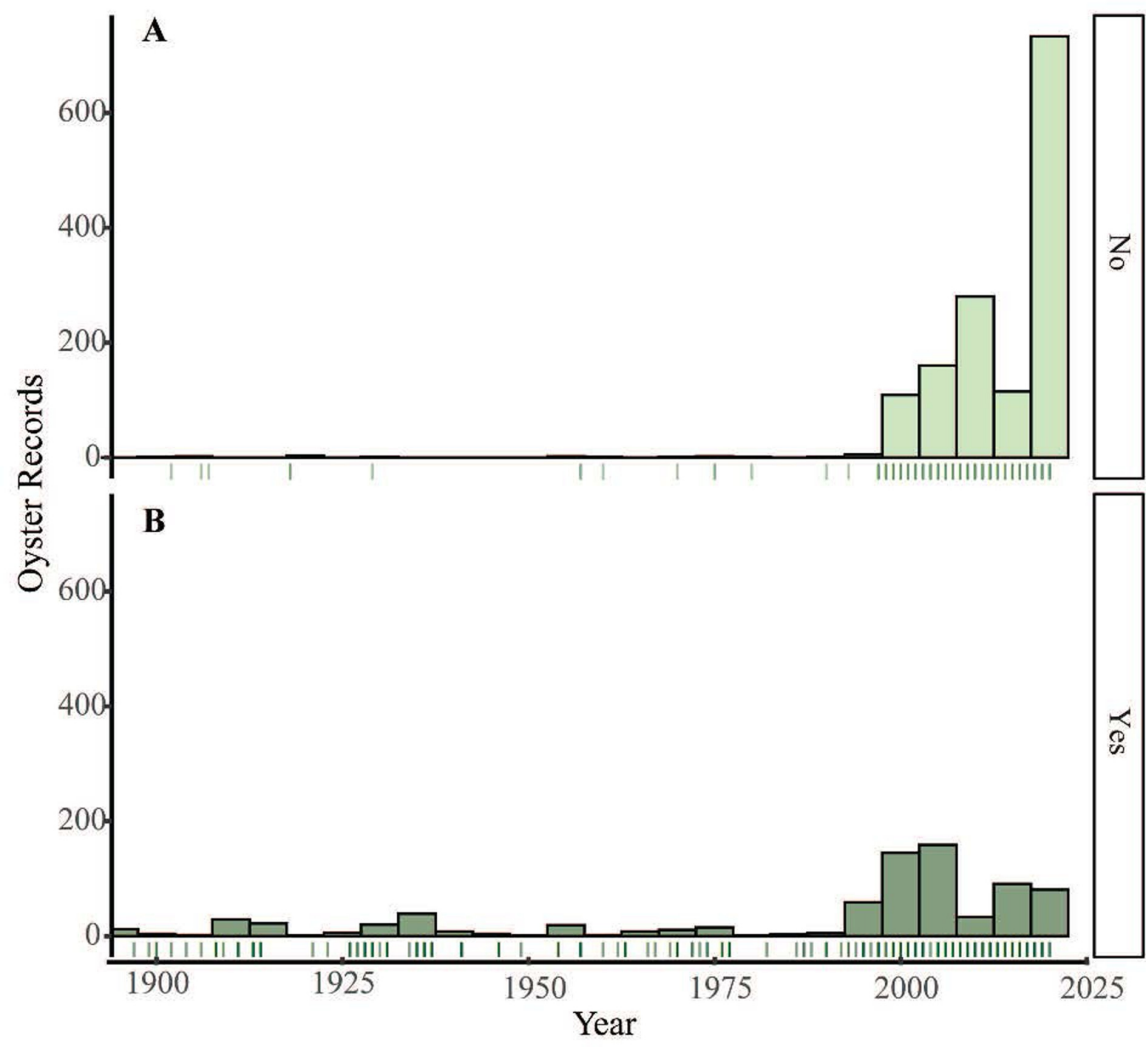
Oyster records over time. (A) unpublished records, which increased after 2000, and especially in 2020 as a result of our crowdsourced effort. (B) published records. This graph omits records before 1900, which consisted of one record from 1602, one from 1804, and 105 from 1840–1899.

The earliest records in our database originate from European expeditions (*e*.*g*., 1602 Vizcaíno expedition noting abundant native oysters in San Diego Bay; 1792 Vancouver expedition with ship naturalist Archibald Menzies observing oysters in Washington). Similar early records likely exist for other estuaries but are difficult to access. We collected 106 records from 1800–1899 and 209 from 1900–1989; there were typically only a few records collected per decade for a total of 318 records during this time span. Collected published and unpublished records increased substantially in the 1990s (Fig 5), and exponentially from 2018–2020 thanks to our data-gathering efforts with experts and from iNaturalist. From 1990–2020, the number of records of oysters increased in most regions, though less so in Canada where regular monitoring led to consistently high relative numbers of records. There was a spike in records from estuaries throughout Southern California during this period largely due to the establishment of a consistent and ongoing oyster population monitoring protocol by author Zacherl’s research laboratory personnel in 2005 that ramped up substantially in 2020 due to increases in research funding (Fig B in S1 File).

#### Change in occupancy of estuaries and distribution

**Fig 6:**
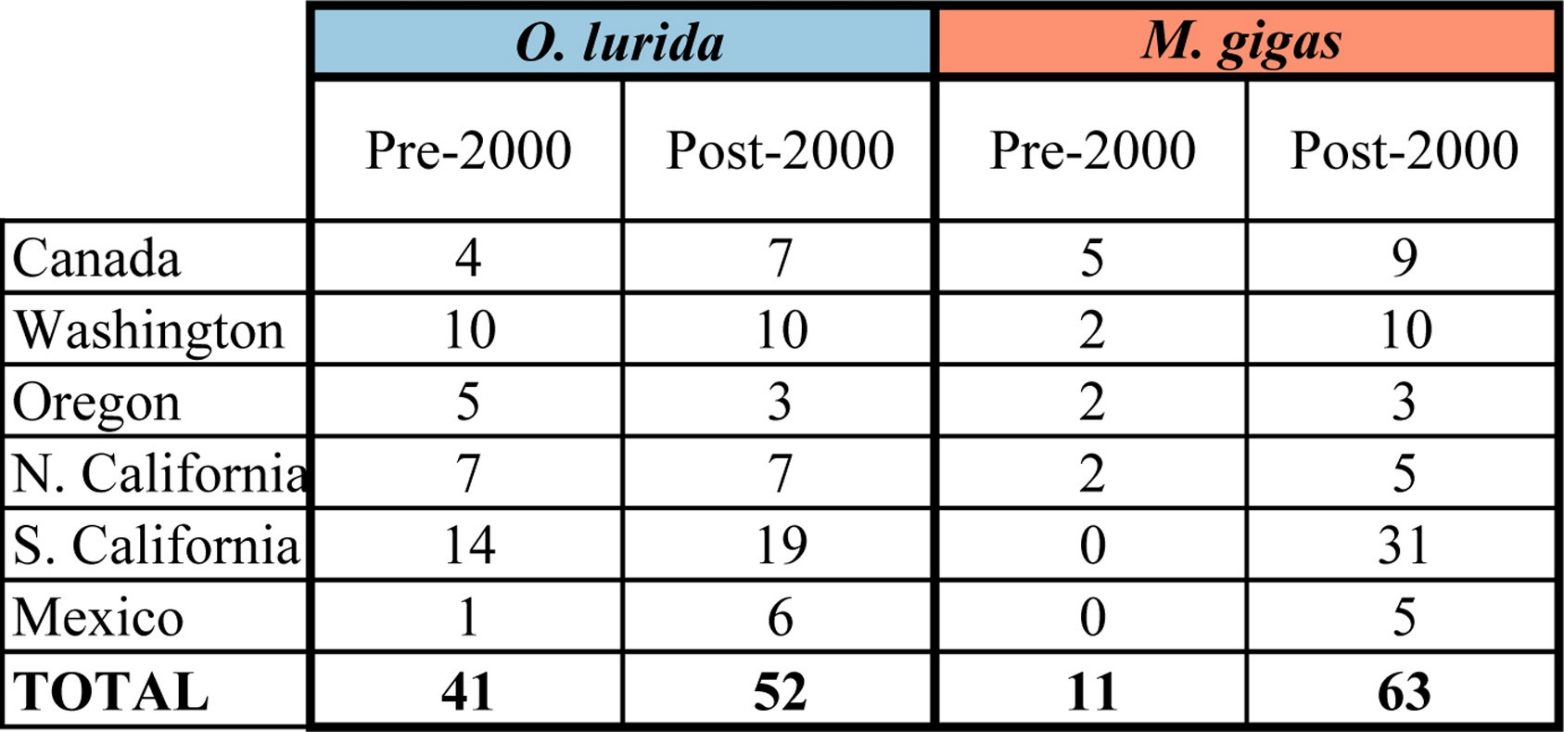
Number of estuaries with oysters present by species, pre-2000 vs. post-2000.

O. lurida. The number of estuaries where *O*. *lurida* was documented as present increased from 45 estuaries pre-2000 to 55 estuaries post-2000 (Fig 6), coincident with greater search effort. Review of the raw data (S3 File) shows that there was no increase in records in the estuaries where they were formerly absent. *O*. *lurida* disappeared from one estuary where it was formerly documented as present, Goleta Slough in Southern California. There are six estuaries where *O*. *lurida* was reported as present pre-2000 but where there were no re-surveys post-2000, so the current population status is unknown (Oregon: Alsea & Tillamook Bays; California: Bolinas Lagoon, Suisun-Grizzly Bay, Bolsa Chica Lowlands, Los Penasquitos Lagoon).

M. gigas. The number of estuaries with the species documented as present increased from 11 pre-2000 to 63 post-2000 (Fig 6). This increase was partly due to previously unsurveyed estuaries being surveyed, but also due to increased presences in places where it was formerly absent. Our database (S3 File) identifies eight such estuaries where the species changed from documented absence to presence, including one in Washington (Juan de Fuca East), three in Northern California (Morro Bay, San Francisco Bay, and Tomales Bay), and three in Southern California (Mugu Lagoon, Newport Bay, and San Diego Bay). In Willapa Bay, Washington, only two records were included in the review before 2000 and, because *M*. *gigas* was documented as absent in both, recent establishment might be concluded, but other evidence supports populations throughout the southern and eastern arms of the bay since 1936 [41]. Repeat surveys did not detect any loss from an estuary where the species was previously reported.

Overall, the northern distribution limits of the two species on this coast are similar (Fig 3) and appear to have remained fairly constant over time. For *O*. *lurida*, latitude 52° 61′ N appears to be the northern limit, and the most northern estuarine complex occupied is the North Coast Fjords. Surveys were not carried out in exactly the same sites, but there is no evidence for any range expansion or contraction at this boundary. For *M*. *gigas*, latitude 53° 41’ N appears to be the northern limit. The most northerly site that was recorded for the species was at Dixon Entrance in 1957, but no recent surveys have been conducted to confirm the species is still there; more recently, Hecate Strait is the most northern estuarine complex where it is documented.

Records of *O*. *lurida* have expanded southward over time, from latitude 30° 36’ S (Bahia de San Quintin) to 26° 80’ S (Estero El Coyote). Since this estuary had not previously been surveyed, it is not clear whether this is a range expansion or simply an expansion of knowledge. It is also possible that the record from this and other southern estuaries is a misidentification, since *O*. *conchaphila* ranges from approximately here southward and the two species are morphologically indistinguishable [13]; genetic analyses are underway to verify these records (J. Lorda pers. comm.). In this part of Mexico, records of *M*. *gigas* have also extended southward, from latitude 32° 55’ S to 26° 76’ S. The most southerly site where *M*. *gigas* was observed in our study is Estero La Bocana. It was previously absent at these latitudes where it is now present. The species also occurs currently further south beyond the scope of our study (J. Lorda, pers. comm.), which focused within the range of *O*. *lurida*.

#### Change in distribution and abundance indices

**Fig 7:**
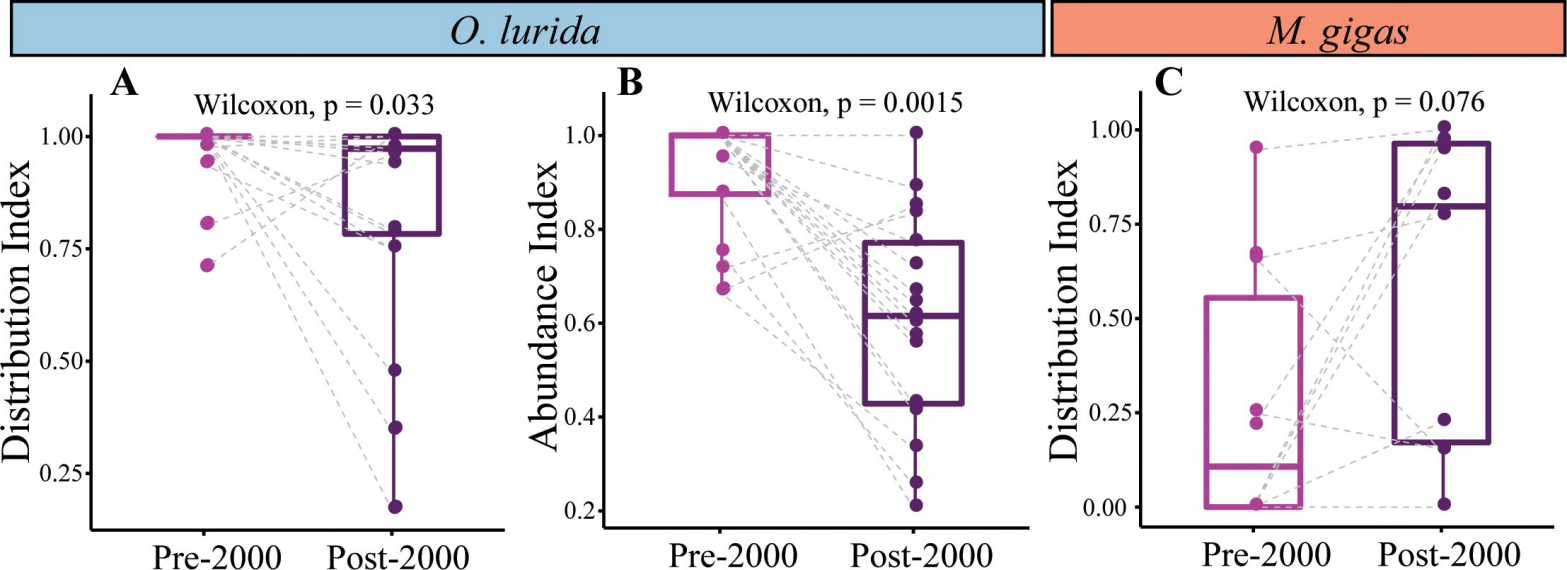
Paired comparisons of distribution and abundance indices in the same estuary over time. (A) *O*. *lurida* distribution index (n = 21 estuaries). (B) *O*. *lurida* abundance index (n = 17). (C) *M*. *gigas* distribution index (n = 10). A dashed line connects the same estuary pre-2000 vs. post-2000.

**Fig 8:**
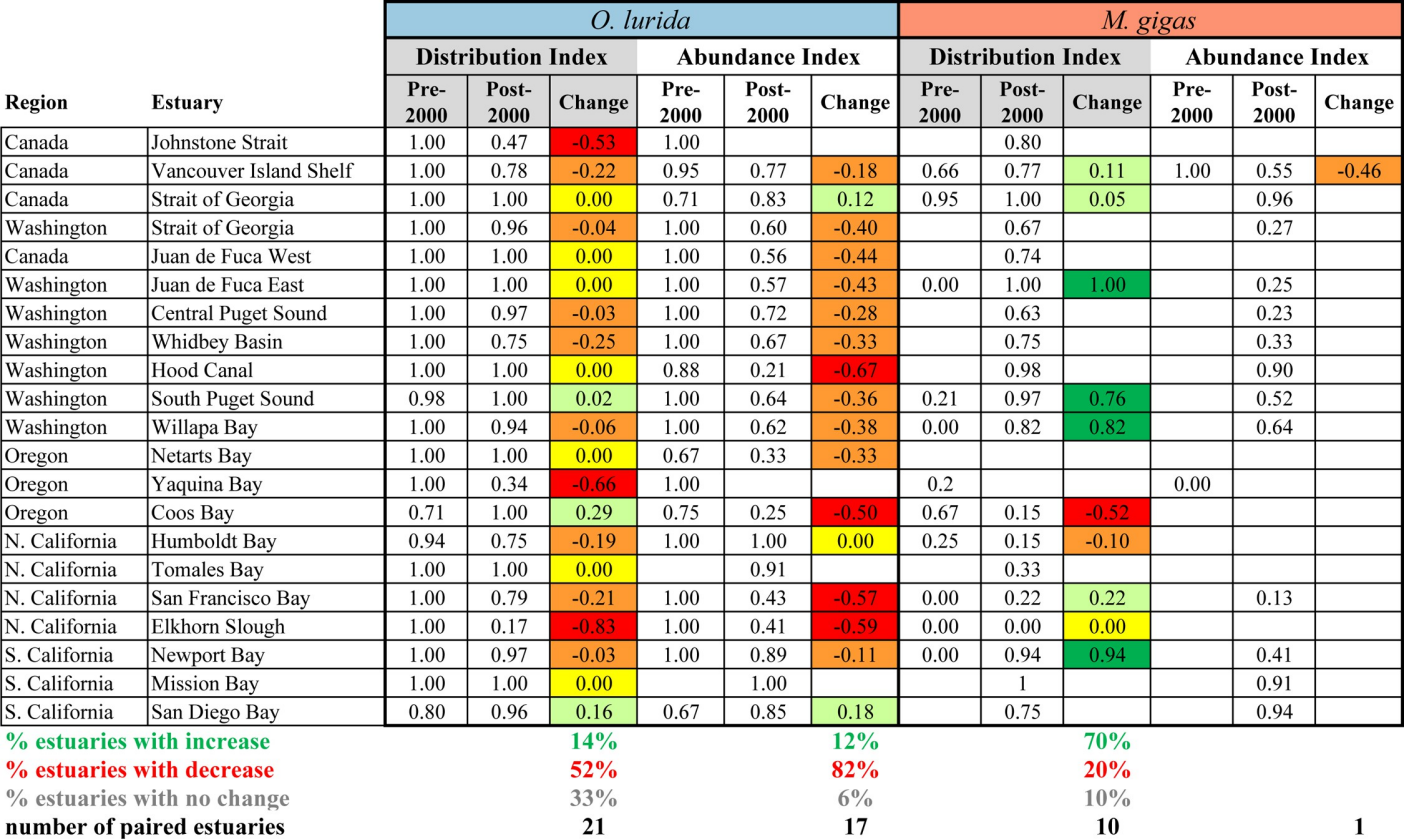
Distribution and abundance indices across estuaries over time. The index is shown for estuaries if at least three records were available for the time period. Estuaries were included in this table only if at least one species had three records for both periods for at least one index. Blank cells indicate that fewer than three records were available. The change in the index at each estuary is shown if data are available for both periods.

For *O*. *lurida*, there was a significant decrease in Distribution Index over time: the frequency of presence vs. absence records within estuaries decreased when comparing records pre-2000 vs. post-2000 (Fig 7). Overall, 52% of estuaries with data for both periods had declines, while 14% had increases. Three of the estuaries had changes in the Distribution Index of ≥0.5; these were all declines, and occurred at one estuary in Canada, one in Oregon, and one in Northern California (Fig 8).

Likewise, *O*. *lurida* showed a significant decrease in Abundance Index over time (Fig 7). We found that 82% of estuaries had declines while only 12% had increases in abundance. Four estuaries had major changes in abundance; these were all declines and occurred at one estuary each in Washington and Oregon and two in Northern California (Fig 8).

For *M*. *gigas*, there was a marginally significant increase in Distribution Index over time (Fig 7). Overall, 70% of estuaries with data for both periods had increases while 20% had decreases. Five of the estuaries had changes in the Distribution Index of ≥0.5; four were increases (three estuaries in Washington and one in Southern California) while one was a decrease (in Oregon) (Fig 8). Changes in the Abundance Index over time could not be calculated because of the near-complete lack of abundance data pre-2000.

### Regional differences

There is large variation in the number of records collected across regions (Fig 4 and Fig B in S1 File): nearly 1,000 records for Canada and Southern California, about half that for Northern California and Washington, fewer still for Oregon, and only a few dozen for Baja California.

There are two very large estuaries with large oyster populations: the Salish Sea (Canada and Washington) and San Francisco Bay (Northern California). Some stretches of coastline have few estuaries with oysters, particularly in Oregon and Northern California (Fig 3). In contrast, Southern California has a high density of small estuaries with oysters (19 with *O*. *lurida* and 31 with *M*. *gigas*); every other region has maximally 10 estuaries with oysters present (S3 File).

**Fig 9:**
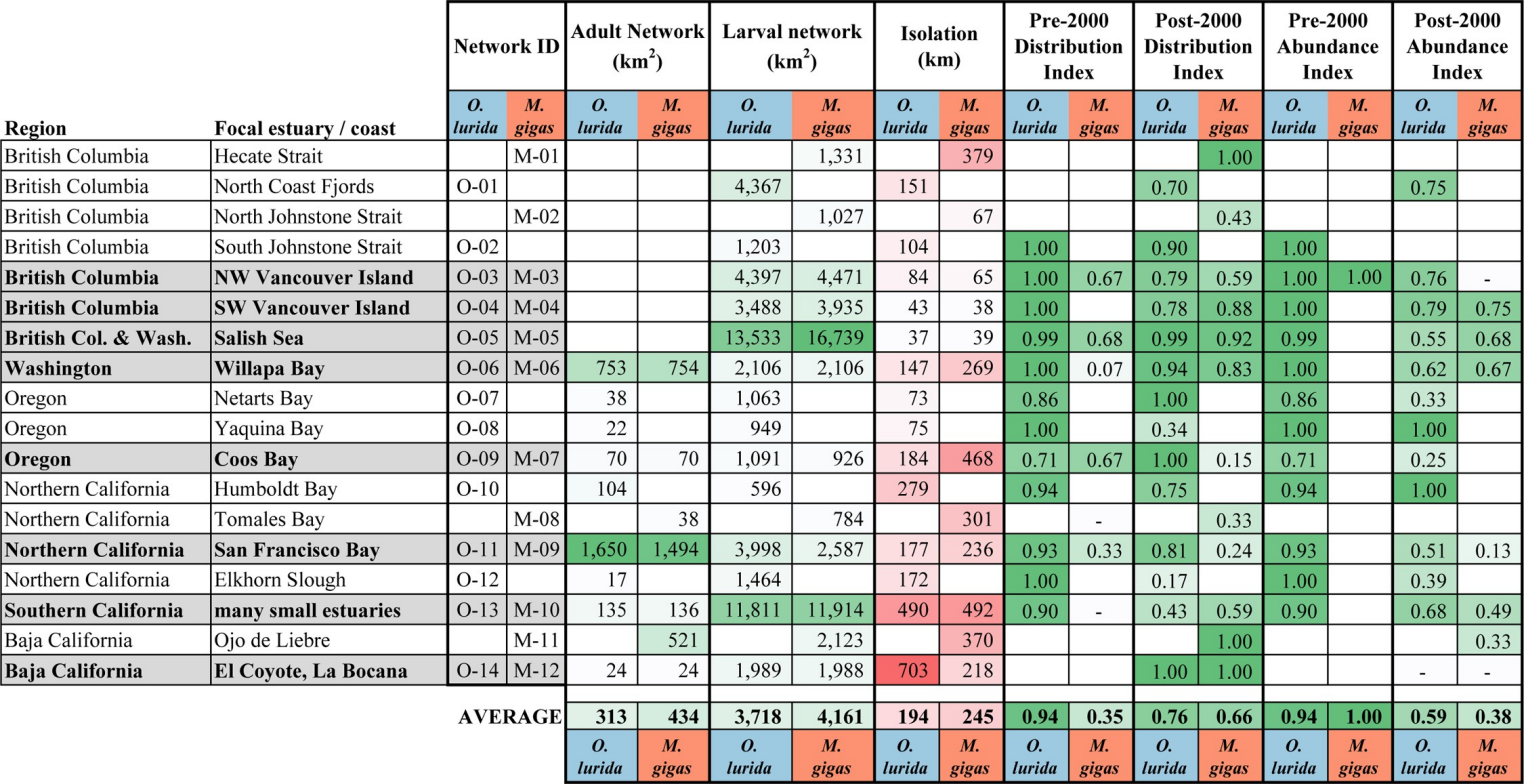
Summary of oyster network data. Networks are listed from north to south. In the eight rows with bold font and gray shading, the networks for both oyster species share the same focal estuary, and can be directly compared. Adult network size could not be calculated for the northern estuaries since no geospatial layer of estuarine boundaries was available. Conditional formatting was applied, in green to parameters that are favorable to oyster population persistence (large networks, high abundance, *etc*.) and in red for network isolation, where high numbers indicate long distances to the next adjacent network, which is unfavorable to among-network population connectivity.

**Fig 10:**
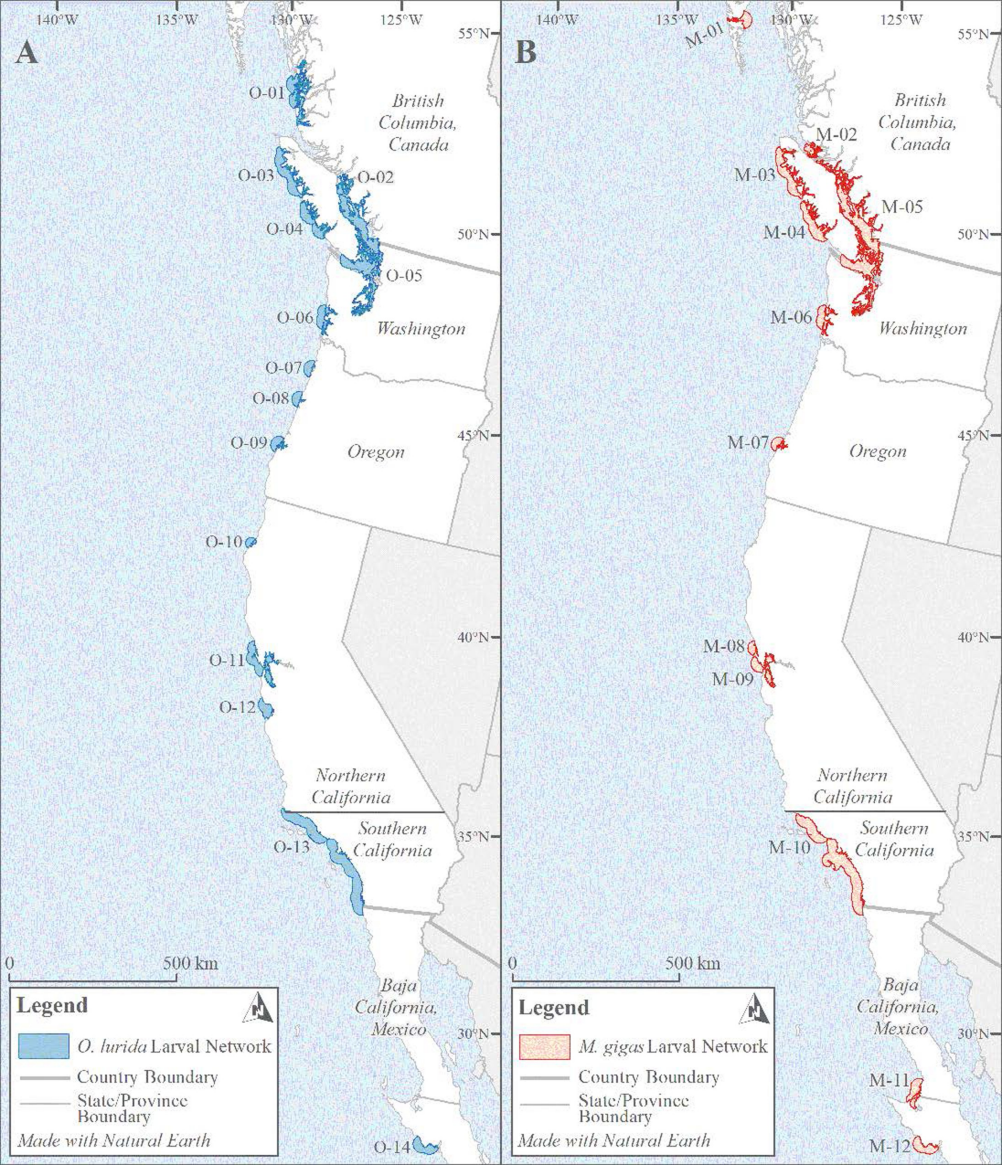
Coast-wide larval networks. Larval networks for *O*. *lurida* (A) and *M*. *gigas* (B) showing large larval networks in the Salish Sea to the north and in Southern California to the south. Networks are similar in size and location between species but not identical.

These regional contrasts are also apparent when comparing networks. San Francisco Bay has an adult network size three times greater than the average along the coast (Fig 9); we were unable to calculate adult network size for the Salish Sea due to lack of estuarine habitat boundary data but clearly the adult network is immense there, too. Larval network size shows somewhat different patterns. The largest larval network, implying high connectivity among sites within the network, for both species is found in the Salish Sea (Fig 9). The second largest larval network is in the southern California bight, where adult network sizes are small, but numerous estuaries are within potential dispersal distances (*i*.*e*., <30 km) of each other (Fig 10). Larval network size is significantly smaller in the central part of the range (Oregon and Northern California) than in the north (Canada and Washington), and marginally smaller than in the south (Southern California and Mexico) for *O*. *lurida* (Fig C in S1 File). Network isolation was lowest in the Salish Sea and Vancouver Island: the northern networks are very near to each other. In contrast, the network in southern California is very isolated (Fig 9); while many small estuaries within this large larval network are connected to each other, the entire network is widely separated from the next to the north and south. Isolation was significantly greater in the southern region than northern or central for *O*. *lurida* (Fig C in S1 File).

**Fig 11:**
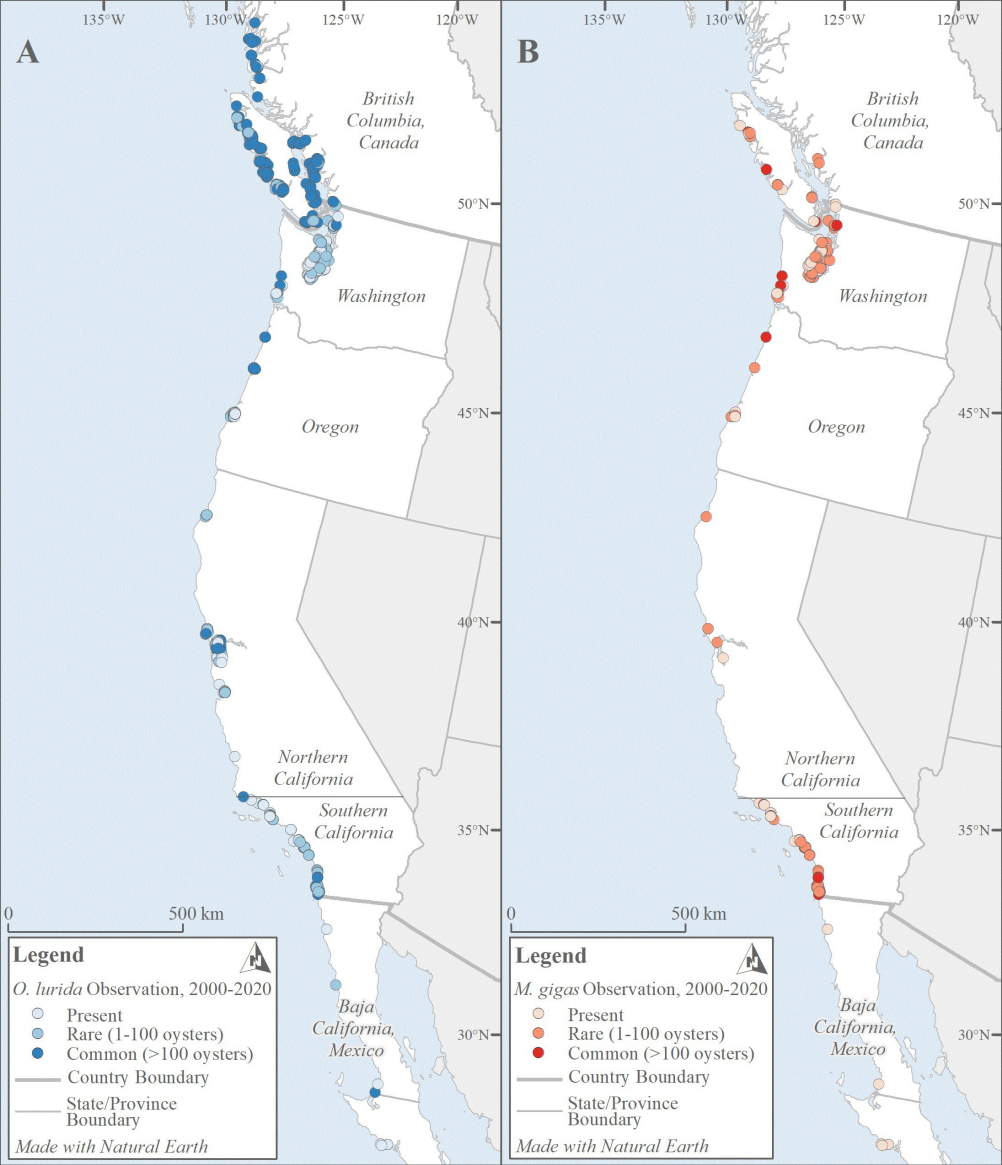
Coast-wide oyster abundance. The abundances of *O*. *lurida* (A) and *M*. *gigas* (B) vary by region. Note that some points may not be visible (*i*.*e*., they are hidden beneath other points) due to map scale.

**Fig 12:**
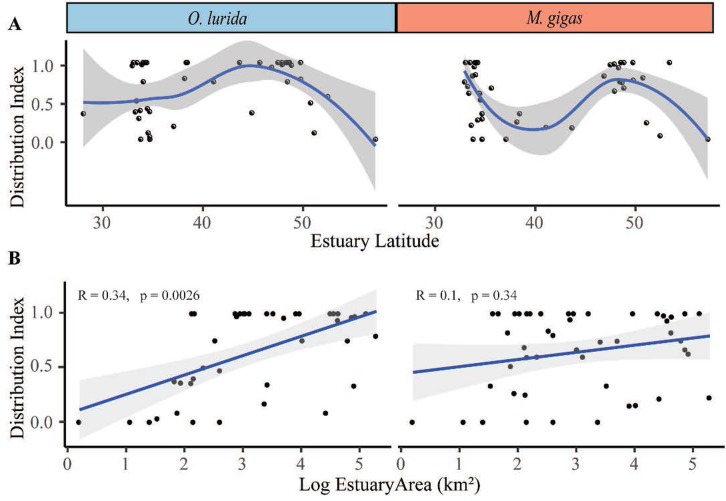
Relationship of estuary latitude and area with distribution index. (A) The distribution index for both *O*. *lurida* and *M*. *gigas* is plotted against latitude using local polynomial regression fitting for this distinctly non-linear relationship. (B) The distribution index for both *O*. *lurida* and *M*. *gigas* is plotted against the log_10_ of the area of the estuary and fitted with linear regression; the Kendall rank correlation is shown. Each point represents a single estuary in all graphs.

In terms of distribution and abundance indices, both oyster species show peak values in the northern part of the range and Southern California (Figs 3 and 11). Particularly striking is the high Distribution Index in Washington for both species (Fig D in S1 File); *O*. *lurida* in particular is present at almost all estuarine sites with information available there. In contrast, distribution and abundance tend to be lower in Oregon and Northern California, as well as Baja California (Fig 8, Fig D in S1 File). Overall, plotting the Distribution Index against estuary latitude reveals a distinctly non-linear relationship (Fig 12A) for both species, with a peak around latitudes 45–50° N. In contrast, the Abundance Index showed no clear pattern when plotted against latitude. The Distribution Index showed a significant positive relationship with estuary area for *O*. *lurida* but not *M*. *gigas* (Fig 12B). The Abundance Index showed no significant relationship (Kendall R < 0.2, p > 0.1) with estuary area for either species.

### Species differences

Overall, very similar numbers of records (with information about presence, absence, and/or abundance) were obtained for both oyster species: 1,624 for *O*. *lurida* and 1,696 for *M*. *gigas* (Fig 4). There were a few hundred more *O*. *lurida* than *M*. *gigas* records pre-2000, but the pattern reversed post-2000.

The distribution of the two species is similar and the northern range limits are at similar latitudes, although *M*. *gigas* was observed slightly farther north (Fig 3). Both species are limited almost entirely to bays and estuaries, but there are a few records of *O*. *lurida* on the open coast of Southern California. *O*. *lurida* is documented as currently present in 52 estuaries while *M*. *gigas* is documented in 63 (Fig 6); most of the estuaries that only have *M*. *gigas* are small ones in Southern California.

**Fig 13:**
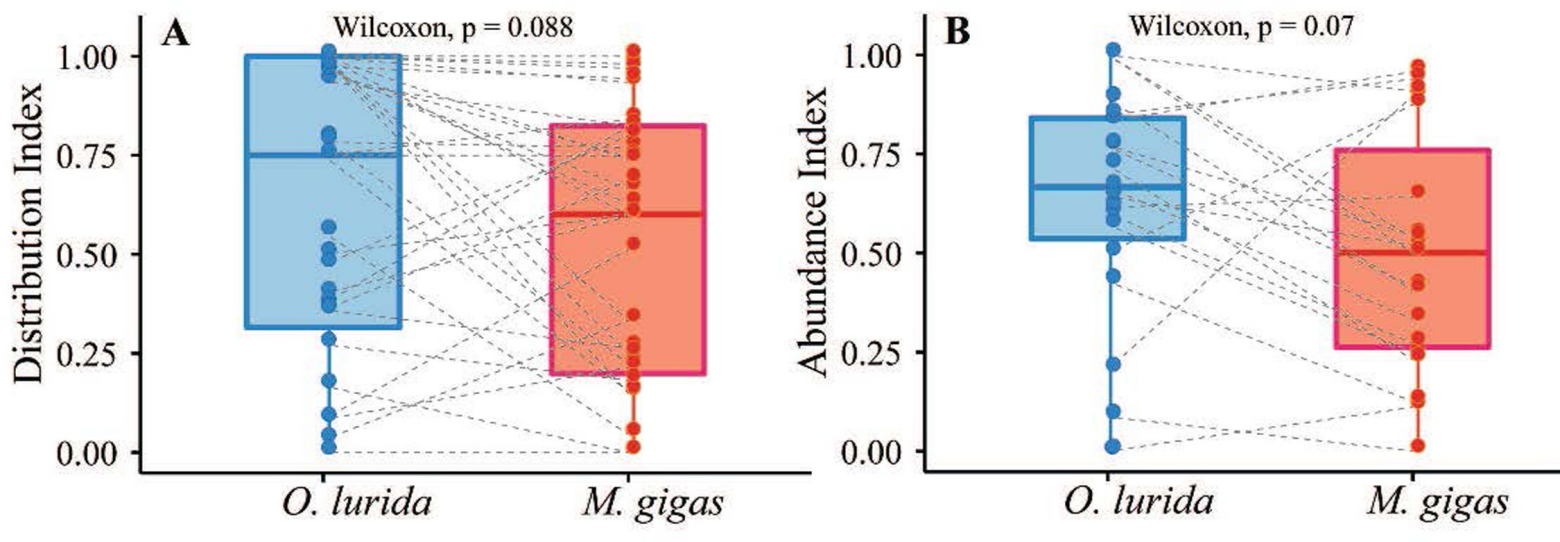
Paired comparisons of distribution and abundance indices between *O*. *lurida* and *M*. *gigas* in the same estuaries. (A) Distribution index (n = 39 estuaries); (B) Abundance index (n = 19). A dashed line connects records of the two species in the same estuary and p value indicates significance of Wilcoxon test.

*O*. *lurida* distribution was fairly stable over time, while *M*. *gigas* increased occupancy of estuaries in Southern California and in Canada post-2000, and the two species underwent opposite trends in Distribution Index (declining for *O*. *lurida*, increasing for *C*. *gigas*). Nevertheless, for the post-2000 period, the distribution and abundance indices remained marginally higher for *O*. *lurida* than *M*. *gigas* in estuaries where both occurred (Fig 13). San Francisco Bay is perhaps the most extreme example of this pattern; *O*. *lurida* has a much higher Distribution and Abundance Index than *M*. *gigas* in this estuary (Figs 3 and 8). The pattern holds for most regions (Fig D in S1 File), though in Canada and southern California the average Distribution index of *M*. *gigas* is higher than it is for *O*. *lurida*. Overall, the distribution indices of the two species are highly correlated across estuaries (Kendall R = 0.52, p < 0.0001, Fig A in S1 File), but the abundance indices are only weakly correlated (Kendall R = 0.21, p = 0.17).

We identified a larger number of networks for *O*. *lurida* (14) than *M*. *gigas* (12). *O*. *lurida* has additional networks in Canada and Oregon not matched by *M*. *gigas*; however *M*. *gigas* has an additional network in Baja California (Fig 10). Network isolation and size of both adult and larval networks were very similar between species (p > 0.9 in paired Wilcoxon tests), but the network was larger in the Salish Sea for *M*. *gigas* and in the San Francisco Bay area for *O*. *lurida* (Fig 9). The sum of the area of all larval networks combined provides an index of coastal habitat being used by the dispersal stage of these oysters; this was 52,057 km^2^ for *O*. *lurida* and 49,932 km^2^ for *M*. *gigas*.

### Current substrates used by oysters

**Fig 14:**
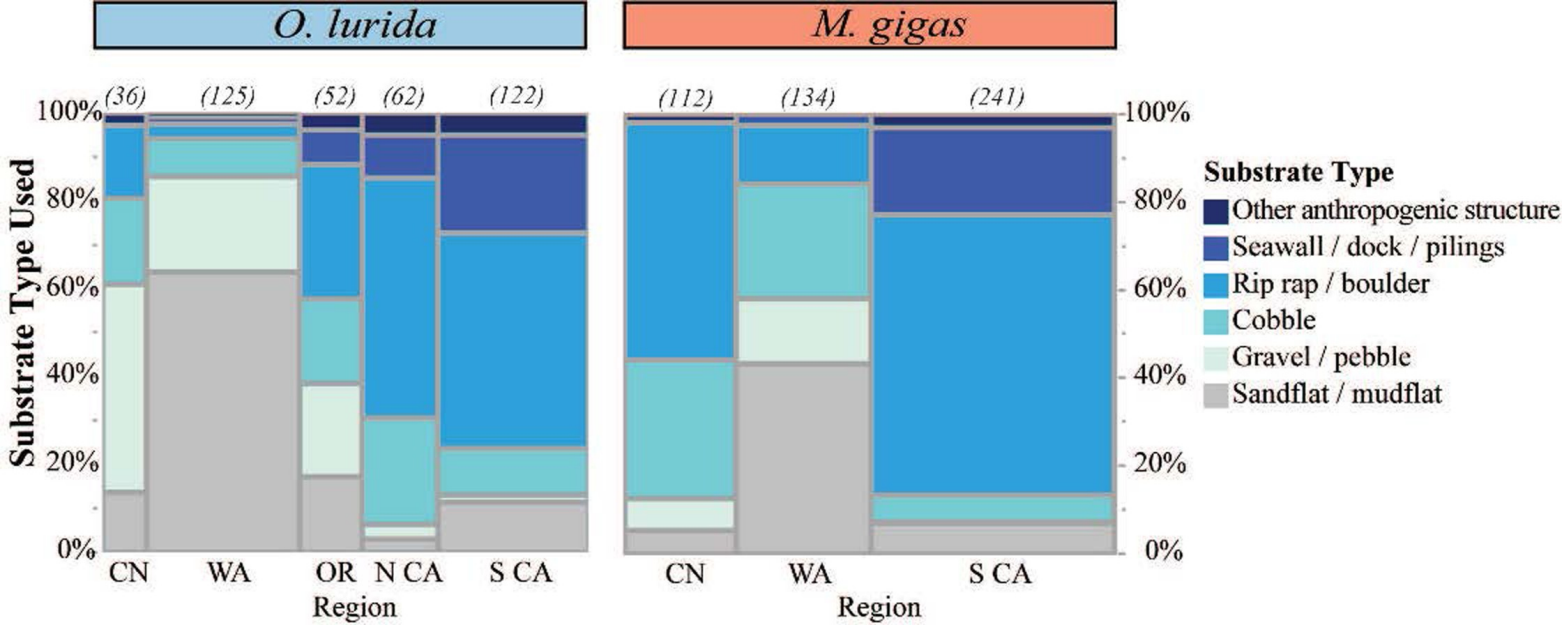
Proportion of substrate types used across regions for both oysters. The number of records for each species and region is shown in parentheses above each column. Abbreviations: CN = Canada, WA = Washington, OR = Oregon, N CA / S CA = Northern / Southern California. Substrate types are arranged from smallest (bottom) to largest (top); details on substrate type definitions are in the Methods.

**Fig 15:**
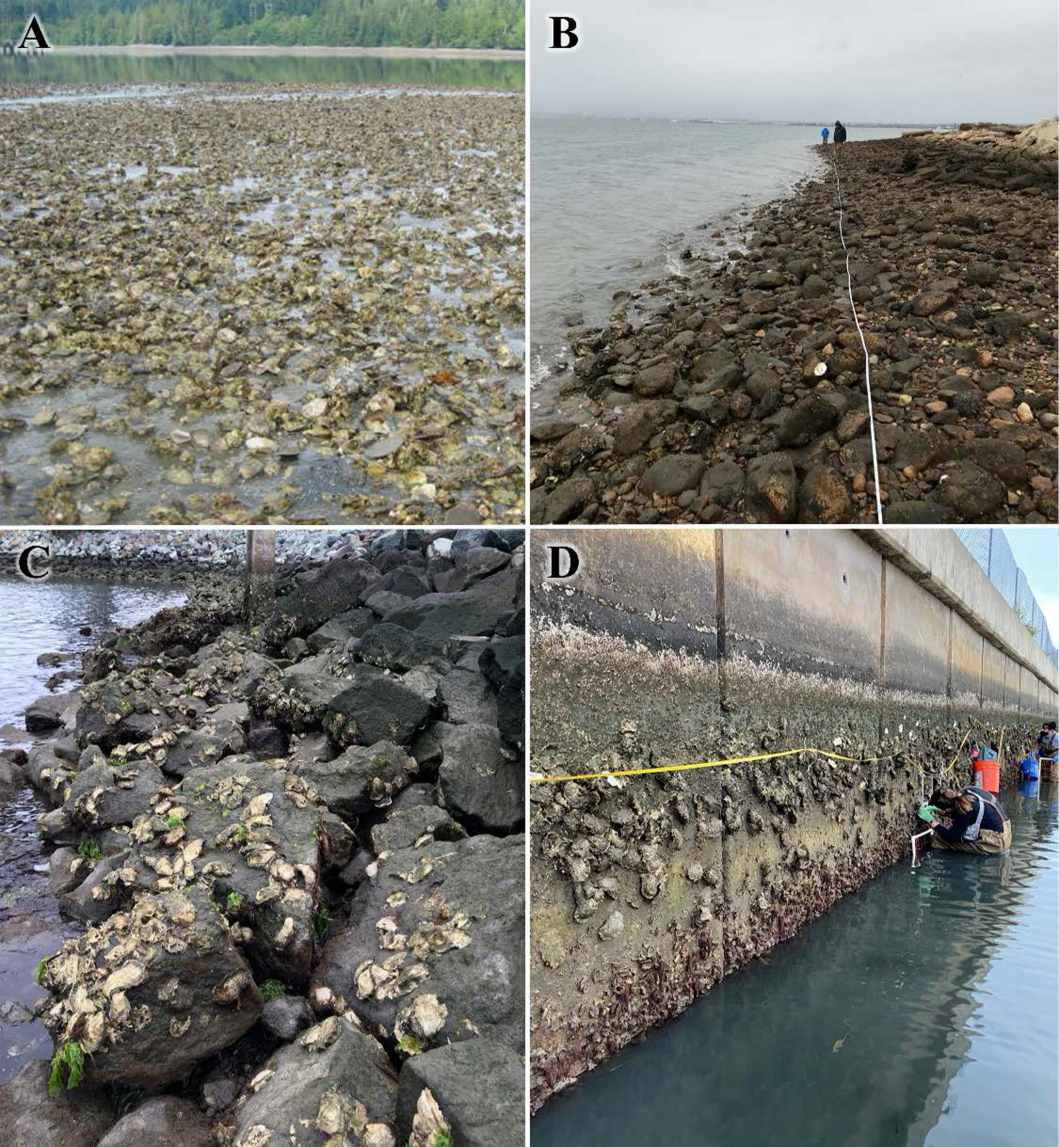
Examples of substrate types in survey. (A) *O*. *lurida* bed in North Bay, Case Inlet, Washington (Photo: B. Blake). (B) *M*. *gigas* on cobble at Chula Vista Wildlife Reserve, San Diego Bay, California (Photo: D. Zacherl). (C) *M*. *gigas* on riprap and pier piling in San Diego Bay, California (Photo: B. Perog). (D) *M*. *gigas* and *O*. *lurida* on seawall in San Diego Bay, California (Photo: D. Zacherl).

Across all regions and both species, oysters were found most commonly on riprap and boulders. Substrate use by *O*. *lurida* differed significantly (chi = 244, p < 0.0001) by region. In Canada and Washington, *O*. *lurida* was found more frequently on smaller substrates such as sandflat/mudflat and gravel/pebble but in the southern regions, *O*. *lurida* was found more commonly on riprap/boulder, with its use of seawalls/docks/pilings increasing towards southern California (Figs 14 and 15). *M*. *gigas* also showed significant differences in substrate use by region (chi = 237, p < 0.001). In Southern California, it was found most commonly on riprap/boulder compared to in Washington and Oregon, where it was most frequently reported on sandflat/mudflat. But in Canada, substrate use was similar to the southern end of the range, with this species most frequently reported on riprap/boulder.

Across all locations, when comparing substrate use directly between the two species, their substrate use differed significantly (chi = 48.57, p < 0.0001, Fig E in S1 File). *O*. *lurida* was found somewhat more frequently on gravel/pebble and sandflat/mudflat relative to *M*. *gigas* which was more common on riprap/boulder.

Differences in substrate use across locations may be due to inherent differences in available substrate. Our investigation of substrate availability vs. oyster use in a northern and southern estuary revealed contrasting results (Fig F in S1 File). In San Diego Bay; both species recruited to habitats in proportions similar to their availability, indicating neither avoidance nor preference (*O*. *lurida*: p = 0.13, FET; *M*. *gigas*: p = 0.06, FET). For both species in Puget Sound, use differed significantly from availability (*O*. *lurida*: p = 0.0180, FET; *M*. *gigas*: p = 0.0031, FET). In Puget Sound, the selection index (*w*_*i*_) indicated that for both species sandflat/mudflat and cobble were recruited onto more frequently than their availability would predict (*w*_*i*_ = 1.36 and 919.60 for *O*. *lurida* and 1.10 and 3251.91 for *M*. *gigas*, respectively), and gravel/pebble, riprap/boulder, and seawall/docks/piling were recruited onto less frequently relative to availability (*w*_*i*_ = 0.59, 0.53, and 0.00 for *O*. *lurida* and 0.55, 0.71, and 0.00 for *M*. *gigas*, respectively). Based upon the 95% confidence intervals, *O*. *lurida* recruited to sandflat/mudflat significantly more than was available, indicating preference, while gravel/pebble and seawall/docks/pilings were used significantly less than was available, indicating avoidance. In contrast, *M*. *gigas* recruited to cobble significantly more than was available. Similarly to *O*. *lurida*, gravel/pebble and seawall/docks/pilings were used less than was available.

## Discussion

### Lessons learned and conservation recommendations for *O*. *lurida*

Overall, this novel compilation of observations from multiple sources has greatly expanded our understanding of the distribution and abundance of *O*. *lurida* across its extensive native range spanning thousands of km along the Pacific coast of North America. By integrating published records, unpublished monitoring data, and iNaturalist records, we were able to characterize spatial patterns much more thoroughly and consistently than earlier coast-wide surveys could accomplish [16]. Below we summarize lessons learned and conservation recommendations for: 1) the state of knowledge; 2) distribution; 3) abundance; 4) substrates; and, 5) networks of this native oyster across its range.

#### State of knowledge

Our synthesis revealed stark contrasts in the state of knowledge about *O*. *lurida* across regions, illustrated by variable numbers of records (Fig B in S1 File). Since 2010, there has been a stark increase of new records collected from southern California. Other regions (northern British Columbia, Oregon, Mexico) have relatively few records and so would benefit from characterizing the status of *O*. *lurida* across more sites. We recommend greater investment by governmental resource management agencies and conservation organizations in surveys, as well as engagement of the public through platforms such as iNaturalist. This will enable future robust characterization of temporal changes in distribution and abundance for both species.

#### Distribution

We generally found *O*. *lurida’s* coast-wide distribution to be fairly stable: neither the northern and southern limits nor estuary occupancy have changed much over the past century. This species has remained present in all except one of the estuaries where it was earlier documented and where there is recent data: it was historically present in Goleta Slough, a small estuary in southern California, but recent searches have shown it to be absent there. There are six estuaries where the species was reported pre-2000 but not since; new searches are needed in these locations to determine if it has persisted (Oregon: Alsea & Tillamook Bays; California: Bolinas Lagoon, Suisun-Grizzly Bay, Bolsa Chica Lowlands, Los Penasquitos Lagoon). However, we documented a significant decline in the Distribution Index across the range of the species, indicating that a lower proportion of sites within estuaries had the species present post-2000 vs. pre-2000. This loss of the native oyster from sites where it previously occurred is of concern and should be addressed through restoration efforts. Temporal changes in Baja California cannot be assessed due to limited historical information. We recommend continued surveys so changes to the northern or southern range limits related to the effects of climate change can be tracked, and gain or loss from sites as well as entire estuaries can be detected.

#### Abundance

We found that abundance has declined in records pre-2000 vs. post-2000 in many places. *O*. *lurida* was scored as rare at many sites where it was formerly common. Note that this bar for “common” is quite low: at the most favorable sites for *O*. *lurida* today, there are >1 million oysters in a 20-m stretch of shoreline. Moreover, conservation of this species is hampered by a lack of knowledge about past abundances, leading to a problem of shifting baselines [42, 43]. From archeological information [44], fossil records [29] and Native American cultural knowledge, it is clear that native oysters formed extensive beds in various locations that were depleted via overharvest by European settlers prior to completion of any quantitative studies [45]. Restoration investment in this species has been fairly modest (about $8M in documented funding for all projects 2000–2019), but has demonstrated success in the immediate restoration footprint [28]. We recommend continuing and even expanding these efforts such that estuary-wide abundance is significantly increased, especially in those estuaries where we have detected the strongest declines.

#### Substrates

Our investigation also provided the first broad characterization of the substrates on which *O*. *lurida* occurs across the range of the species. Since most estuaries on this coast are dominated by soft sediments, oysters in natural estuaries in the past would have been limited to settling on each other or small bits of gravel or shell. Prior to European settlement of this coast, native oysters formed extensive biogenic beds in various estuaries, but these were mostly depleted by the early 1900s [17, 46]. These beds form when oysters settle on live or dead oyster shells, and may take centuries to form [47]. Our compilation of data on current substrate use showed that in Washington, *O*. *lurida* is still commonly found on sandflats or mudflats, and in fact, its use of such soft-sediment habitats exceeds availability. However, such natural beds are now rare in much of the southern portion of the range. There are likely multiple explanations for the lack of oysters on mudflats in the South. In part, human activities have greatly increased the availability of larger hard substrates such as riprap, docks, and pilings, and these ubiquitous substrates are used by oysters. In addition, mudflats in more developed estuaries have likely been subject to increases in the depth of unconsolidated sediments due to more organic matter that builds up from nutrient-loading and eutrophication. The consequences of these habitat alterations are that oysters cannot survive on mudflats unless they are on large, hard substrates [48]. While it is important to recognize the importance of artificial hard substrates in supporting native oysters in many California estuaries, true restoration would involve bringing back at least some representation of natural biogenic habitat, where oysters are settled upon each other on mudflats. Restoration of biogenic beds is an explicit goal in Washington state [49], but we recommend that it be considered as a restoration goal for at least some locations in all regions.

#### Networks

Our analysis was the first attempt to characterize local oyster networks across the range of *O*. *lurida*, defining areas where adults live close enough to form a regularly interbreeding population, and where larvae can typically be transported among adult locations. The interactive online maps we produced (S4 File) provide local and regional stakeholders key information about network location, size, and isolation and reveal striking differences among regions. In some parts of the coast, such as the Salish Sea and Southern California Bight, native oysters are found in large networks, and in the north, these networks are not far from others. In other parts of the coast, such as Northern California (other than the San Francisco Bay area) and Oregon, oysters occur in small networks, typically isolated from the nearest neighboring network by hundreds of kilometers. The connectivity patterns we found in this spatial analysis correspond generally to those elucidated by a recent genetic analysis of the species [50] (Fig G in S1 File). We recommend that future conservation efforts focus on enhancing networks and network connectivity. Network size can be enhanced by restoring oysters to additional sites within a larger footprint than currently occupied, but within “easy” dispersal distance of larvae. This is especially vital for the smallest networks on the coast (*e*.*g*., O-7, O-8, and O-10, Fig 10). Furthermore, there were some oyster records that were so isolated (*i*.*e*., only a single record beyond 30 km from the next nearest record) that we did not include them in any network (*e*.*g*., single records of *O*. *lurida* in Morro Bay, California and Estero de Punta Banda, Mexico). These are high priorities for additional study (to determine whether there are more sites nearby, which is a possibility for poorly characterized regions) and for potential network enhancement.

In the future, the spatial dynamics of this species could be explored through further analysis using our data, as well as data and tools produced by other entities. For example, our analysis of larval networks examined only the potential likely larval travel distance using tools available in ArcGIS Pro 2.7, and did not model the impacts of ocean currents, temperature, salinity, or other factors that are likely key drivers.

### Spread of non-native oyster and management implications

Our analyses revealed that *M*. *gigas* has a generally similar distribution and abundance to *O*. *lurida* across the range of *O*. *lurida*. This is remarkable because *M*. *gigas* was first introduced to the North American west coast in 1902 (reviewed by [51]), while *O*. *lurida* has occupied locations throughout its range since at least the mid-Pleistocene, 12,000 to 2.5 million years ago [15, 29, 44, 52]. Now, the two species share similar network sizes and levels of isolation, so it is perhaps not surprising that the distribution indices of the two species are highly correlated; estuaries that have one species at multiple sites also tend to have the other species. However, their abundance indices are only weakly correlated and this may be due to a number of factors. *M*. *gigas’* more recent introduction may mean that population densities and dynamics have not yet stabilized. Furthermore, so far there is only limited evidence that the two species may directly interact with or impact one another [24, 53], and it has now been well-established that the species exhibit a zonation pattern (in Washington and California, where they are most common) with only little overlap; *M*. *gigas* occupies an elevational position in the mid- to high-intertidal versus *O*. *lurida* achieving highest densities in lower intertidal to subtidal [19, 26].

Our data reveal that *M*. *gigas* presence in estuaries and abundance across estuaries has already increased over time, particularly in southern California and Washington. In fact, the non-native species is currently present in more estuaries (63 versus 52) across the geographic range of *O*. *lurida* than is the native species. In many estuaries where *M*. *gigas* is now reported, *M*. *gigas* aquaculture has operated at some point, so it is likely that spread and establishment of feral populations resulted from this source [54] or from occasional outplantings by local landowners hoping to ‘farm’ their own oysters, as has been witnessed in San Francisco Bay (E. Grosholz, personal observation). However, in southern California, aquaculture has only occurred for significant time in the Santa Barbara channel and in Agua Hedionda lagoon, while it has established much more broadly throughout southern California estuaries where aquaculture never existed. Indeed in a U.S. west coast estuaries survey published in 1991, *M*. *gigas* was not detected in any southern California estuaries south of Morro Bay [55]. While initial establishment in this region may have been from unintentional introductions from aquaculture facilities or local landowners, this region is also the location of two of the busiest ports along the North American west coast (San Diego and Los Angeles), with shipping activity another likely vector of introduction [56]. Regardless of the source of introduction, the synergy of a high level of connectivity among southern California estuaries (*e*.*g*., large larval networks) and recent warming waters [57, 58] have generated ideal conditions for further *M*. *gigas* proliferation. Their extent is likely to increase even more with climate change, as has been documented along the Northwest European Shelf [59]. Given the pervasive range extension of this species on a global scale, its spread along the North American west coast seems to be a foregone conclusion. Thus, there is a pressing need to integrate this species into regional and local conservation planning and management. The virtues of non-native species ecosystem services have been debated extensively elsewhere [54, 60, 61]; it is clear that resource managers along the North American west coast will now by necessity enter into the debate. In some instances where the services provided by *M*. *gigas* are considered desirable, expansion of the non-native species may be welcomed. But in many areas, where conservation and restoration of the native species is a priority, habitat restoration should explicitly be designed to decrease representation of the non-native, such as by deploying settlement substrates lower in the intertidal where the native is more common [19] or by manipulating habitat rugosity, composition, and orientation of substrates to favor recruitment of the native relative to the non-native [62].

In terms of documenting *M*. *gigas* spread, it is striking that most of the records of its presence were from unpublished records and iNaturalist (84% of records compared to 44% for *O*. *lurida*). This again may be a function of the more recent introduction of *M*. *gigas*, such that publications of its presence are lagging its explosive spread. Also contributing to the stark difference in source of records among species may be the fact that *M*. *gigas* is more obvious (significantly larger) and located in the middle to upper intertidal (thus more detection time) whereas *O*. *lurida* is found at a lower tidal elevation [19] and more strongly prefers to recruit underneath surfaces [63], making detection much more challenging. But this also points to the importance of community science in detecting the proliferation of this non-native species. Harnessing the power of community-sourced data enabled detection of *M*. *gigas’* spread in our study, and we encourage other scientists to harness the power of this underutilized source of data moving forward.

### Applications to other species

While the motivation for our investigation was conservation of *O*. *lurida* on the Pacific coast, the project yielded lessons that are applicable more broadly. We briefly highlight three of these below, 1) the value of range-wide spatial ecology for conservation planning, 2) the use of GIS tools and analyses to characterize local networks and connectivity, and 3) the relevance of considering similar non-native species along with focal native species.

Our mapping of distribution and abundance of a native oyster across its entire range yielded novel insights that were not evident from previous characterizations at the level of individual estuaries or smaller regions. For conservation organizations or funding organizations that operate at a large scale, a range-wide assessment can reveal critical areas, with the biggest returns for conservation investment. In our case, the study revealed that Mexico, northern California and Oregon appear to have the most imperiled oyster populations, and yet an earlier synthesis revealed that these areas have had little investment in restoration [28]. This finding will spark future investments. Range-wide recovery plans are typically generated for species legally recognized as endangered at a national or international scale, though the quality of such plans could be improved [64]. For terrestrial systems, there has been an emerging recognition of the importance of thoughtful range-wide strategic planning [65–67]. Such large-scale conservation planning across the range of marine species, especially invertebrates, remains rare. Based on how valuable it proved for our system, we recommend expansion of range-wide species conservation assessments and prioritization.

We developed and implemented novel spatial analysis approaches that yielded an enhanced understanding of conservation issues. We used relatively new ArcGIS Pro tools including Distance Accumulation (to generate polygons encompassing local larval networks) and Optimal Region Connections (to calculate connectivity among them). The resulting analysis revealed stark contrasts in network size and connectivity among regions. These are critical findings, given the importance of metapopulation dynamics for oysters [68] and other species. We recommend broader adoption of such spatial analysis approaches to spatial ecology analyses to support network connectivity analyses that can be critical for conservation [69–71]. Such landscape ecology approaches remain rare for marine foundation species, but our results illustrate their utility.

An unexpected finding of our investigation was the similarity in distribution between two quite distantly related oyster species, the native *O*. *lurida* that was our focus and the non-native *M*. *gigas*. Abundance, network size and connectivity peaked in the same regions for both species. This makes clear that any conservation planning for the native species must proceed with consideration of the non-native one. Perhaps the non-native species serves similar functions to the native and can replace these functions if it increases in abundance in places where the native is rare [60]. However, numerous negative impacts must also be considered [54]. We thus recommend that spatial ecology analyses of native species of concern be paired with analyses of overlapping non-native species, so that conservation planners can evaluate the data and explicitly consider which species and functions they value, deciding whether to focus on conservation of native species and/or whether to embrace novel ecosystems [72, 73].

### Spatial ecology using crowd-sourced data

We used a crowdsourced database approach to integrate records from the published literature and iNaturalist entered by trained student interns with data input by researchers and resource management agencies. In just a few months during the beginning of the COVID-19 pandemic we were able to compile orders of magnitude more data on oyster distribution and abundance on this coast than previous syntheses that took years to compile [16].

In the past decade, there has been a proliferation of what is often called “community science;” the crowd-sourced data that have been collected through such efforts has been widely analyzed by scientists, especially for terrestrial vertebrates [74, 75]. A comprehensive review of monitoring in Europe suggested volunteer monitoring can yield excellent assessments of biodiversity, so long as the spatial/temporal sampling frequency is high and protocols are robust [75]. This type of model has rarely been used for marine systems and habitat-forming species. iNaturalist is the most widely used multi-taxon, on-line database for community wildlife monitoring. It holds promise for assessing more obscure marine and rare species because there are many contributors cataloguing entries in many places and a relatively low technological/scientific threshold for participation [76].

We found that coordination, training, and quality control were key to developing a high-caliber database. We provided written guidance and webinar-based training to all collaborators and designed the data input methods, including the Portal, to be straightforward so that everyone used consistent definitions (*e*.*g*., for abundance categories and substrate types) and data input techniques. Incorporation of iNaturalist records into the database substantially increased understanding of oyster distributions, especially for *M*. *gigas*. However, since there were known species misidentifications in records from iNaturalist, it was critical that trained eyes review all iNaturalist records and confirm data accuracy using photos associated with those records. This led to the culling of many probably correctly identified records but was deemed necessary to preserve the quality of the overall database, particularly when dealing with such morphologically plastic species as oysters.

We conclude that partnerships between community scientists and monitoring organizations can yield rich datasets [77]. Community science can contribute to spatial ecology and conservation, even of less charismatic groups like invertebrates [78]. Our assessment of spatial and temporal dynamics of a native and a non-native oyster on the west coast of North America can serve as a model for spatial ecology of other marine foundation species whose distribution is poorly known. By bringing together diverse sources of information in a rigorous framework, from early explorer journals to unpublished government surveys to internet-based community observations, we can better understand and conserve critical coastal species and habitats.

## Supporting information

S1 File Additional detail on methods and results. (PDF)

S2 File Instructions participants were provided for the ArcGIS online portal. (PDF)

S3 File Raw records, metadata, and summary of records by estuary. (XLSX)

S4 File Interactive ArcGIS on-line map—the “the Olympia & Pacific oyster data portal”—on the NOAA GeoPlatform. (DOCX)
