# Supplementary material for: Mapping oysters on the Pacific coast of North America: A coast-wide collaboration to inform enhanced conservation
Source: PLoS One. 2022 Mar 17;17(3):e0263998. doi: 10.1371/journal.pone.0263998 (PMC8929589; doi:10.1371/journal.pone.0263998)

SUPPLEMENTAL FILE 1 - ADDITIONAL METHODS AND RESULTS
For Kornbluth *et al.* 2021, Mapping oysters on the Pacific coast of North America: a coastwide collaboration to inform enhanced conservation.

Table A. Attribute data for each record in the mapping database.

Data	Type of information	Detail on data entered	Required?
Location of site	Latitude & longitude	Decimal degrees	X
Ostrea lurida abundance	Estimated number of live oysters in 20-m linear stretch of shoreline	Absent (0), Rare (1-100), Common (>100), Present but abundance unknown; Not Known (whether present or not)	X
Magallana gigas abundance	"	"	X
Unidentified oyster abundance	"	"	X
Observer	Name		X
Time	Year of observation		X
Source	Origin of observation	Published data, long-term monitoring, personal observation, iNaturalist, other	X
Citation	(if published record)	Full citation	
Sensitive data	Yes or no	If yes, records removed from publicly shared data but included in analyses	
Site name		Any descriptor desired	
Substrate	The most common substrate to which live oysters are attached	Riprap or boulder (>25 cm), cobble (5-25 cm), gravel/pebble (0.6-5 cm), sandflat/mudflat, seawall/dock/piling, other anthropogenic structure	
Notes	Any other information	Quotations from historic literature included as appropriate	
Attachments	Photos, references, *etc.*		

Table B. Portal geospatial reference data layers.

Layer	Source	Type	Purpose
Continually Updated Shoreline Product (CUSP)	National Oceanic and Atmospheric Administration (NOAA) National Geodetic Survey: https://shoreline.noaa.gov/data/datasheets/cusp.html	Map service	Provides the most current tidal-datum referenced shoreline representation of the United States and its territories using NOAA and non-NOAA contemporary sources to update our nation's dynamic shorelines. (n.d.)
Protection Status by GAP Status Code	U.S. Geological Survey, Gap Analysis Project (GAP). Protected Areas Database of the United States (PADUS), V2.0 Combined Feature Class: https://services.arcgis.com/P3ePLMYs2RVChkJx/arcgis/rest/services/Protection_Status_by_GAP_Status_Code/FeatureServer/0	Map service	A measure of management intent to permanently protect biodiversity. GAP 1&2 areas are primarily managed for biodiversity, GAP 3 areas are managed for multiple uses including conservation and extraction, GAP 4 areas have no known mandate for biodiversity protection. Shows the full range of GAP Status Codes (1-4) for all lands and marine areas. (2018)
West Coast USA Estuarine Biotic Habitat	Pacific Marine and Estuarine Fish Habitat Partnership, Pacific States Marine Fisheries Commission GIS, Oregon Coastal Management Program: https://honu.psmfc.org/server/rest/services/PMEP/West_Coast_USA_Estuarine_Biotic_Habitat/MapServer	Map service	These data represent the Biotic Component (BC) of the Coastal and Marine Ecological Classification Standard (CMECS) for estuaries of the West Coast of the contiguous U.S. (2020)
West Coast USA Eelgrass Maximum Observed Extent	Pacific States Marine Fisheries Commission: https://psmfc.maps.arcgis.com/home/item.html?id=12ed43ed0fe342bc86225268cbb638c7	Map service	This layer represents the maximum observed extent of eelgrass (*Zostera sp.*) habitat on the U.S. West Coast, based on the best available existing spatial data showing the current and historic extent of eelgrass in the region. (2018)
"NatOysPres" Washington state Oyster Presence	Washington Department of Fish and Wildlife (WDFW)	Shapefile provided by WDFW	The NatOysPres dataset displays native oyster known current and predicted presence in Washington by shoreline segments as adapted from the Washington Department of Natural Resources ShoreZone Inventory. Presence, known or predicted is one or more oysters per shoreline segment." (2001)
Native Olympia Oyster Collaborative (NOOC) Restoration Projects	NOOC, Elkhorn National Estuarine Research Reserve, and Tijuana River National Estuarine Research Reserve, in collaboration with several organizations and funded by the National Estuarine Research Reserve Science Collaborative: https://services2.arcgis.com/C8EMgrsFcRFL6LrL/arcgis/rest/services/NOOC_Native_Olympia_Oysters_Collaborative_Restoration_Projects_renamed/FeatureServer	Map service	Over the past decade, an increasing number of restoration projects have been conducted at various sites from British Columbia to Baja California. In 2018, with funding from the National Estuarine Research Reserve Science Collaborative, a thorough survey was conducted to obtain information on past restoration projects. All project leads generously shared information and photos about their project, making this synthesis possible. This layer represents the location of the Olympia Oyster restoration projects included in this synthesis. (2019)

Table C. Geospatial data used to create seamless I: coastline/land and II: estuary/ecosection boundaries.

**I. The merged coastline/land boundary polygon was created using:**		
**Location**	**Source**	**Description**
**British Columbia, Canada**	Ocean - TRIM Enhanced Base Map (EBM), Ministry of Forests, Lands, Natural Resource Operations and Rural Development: https://catalogue.data.gov.bc.ca/dataset/905940c3-284b-4bba-8669-b2bf855542ca	Ocean.TRIM-EBM is a 2D product containing TRIM 1 features excluding toponymy, contours, and DEM for which area based features have been closed to form polygons, symbol points have been replaced with polygons and vertical integration of streams has been removed. (2014)
**Alaska, Washington, Oregon, & California, U.S.**	Continually Updated Shoreline Product (CUSP), National Oceanic and Atmospheric Administration (NOAA): https://shoreline.noaa.gov/data/datasheets/cusp.html	Provides the most current tidal-datum referenced shoreline representation of the U.S. and its territories using NOAA and non-NOAA contemporary sources. (n.d.)
**Baja California, Mexico**	Instituto Nacional de Estadistica y Geografia (Mexico; available through Stanford University): https://earthworks.stanford.edu/catalog/tufts-mexicowaterbodies10	This polygon dataset shows major waterbodies, including the coastline. (2010)
**II. Estuary & physical ecosection boundary polygons were created using:**		
**Location**	**Source**	**Description**
**British Columbia Marine Ecosections**	British Columbia DataBC: https://catalogue.data.gov.bc.ca/dataset/marine-ecosections-coastal-resource-information-management-system-crims	This dataset identifies the 12 Provincial marine ecosections on Canada's Pacific Coast. (1994)
**U.S. estuaries (except Puget Sound)**	Pacific States Marine Fisheries Commission: https://www.arcgis.com/home/item.html?id=29d63729a5d64e74a9c300e71ca29034	This layer represents the current and historical tidal wetlands, or estuary extent, for the West Coast of the contiguous United States. Map Image Layer from Pacific States Marine Fisheries Commission. (2017)
**Process step:** Created Abbott's Lagoon, UCSB Campus Lagoon, Santa Barbara Harbor, and Redondo Beach estuary polygons by hand digitizing using World Imagery as base layer.		
**U.S. Salish Sea/Puget Sound Basins**	Washington Department of Fish and Wildlife: https://erma.noaa.gov/northwest/erma.html#/x=-	The Salish Sea (Map of the Salish Sea & Surrounding Basin, Stefan Freelan, WWU, 2009) includes the Strait of Georgia, Desolation Sound, The Strait of Juan de Fuca, and Puget Sound, which is further

	123.44039&y=48.39419&z=8&layers=16+7531	classified into these marine basins: Hood Canal, Main Basin (Admiralty Inlet and Central Basin), South Basin, and Whidbey Basin. (2015)
**Process step:** Puget Sound Basins and British Columbia Salish Sea ecosections were clipped at the U.S./Canada boundary and merged.		
**Baja California, Mexico**	Instituto Nacional de Estadistica y Geografia (Mexico; available through Stanford University): https://earthworks.stanford.edu/catalog/tufts-mexicowaterbodies10	This polygon dataset shows major waterbodies in Mexico. (2010)
**Process step:** Created Baja California estuary/ocean boundaries by hand digitizing using World Imagery as base layer, editing points at estuary mouths.		

Figure A. Relationships among indices.
The two indices are plotted against each other for (A) *O. lurida* and (B) *M. gigas.*
The two species are plotted against each other for (C) Distribution index and
(D) Abundance index. Each point represents an estuary.

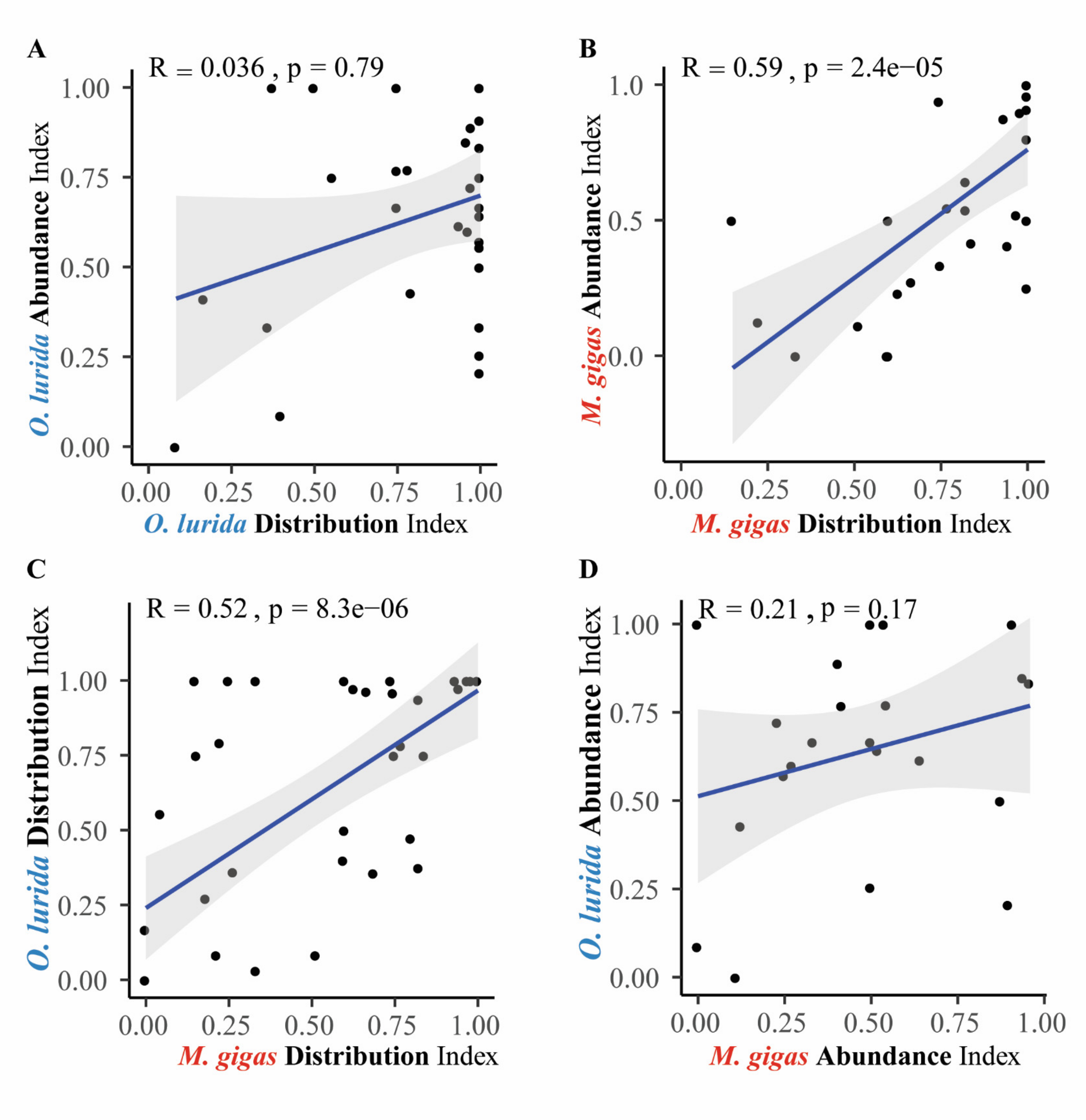

Figure B. Oyster records since 1990 across regions. Note that southern California (S. Calif.) had over 300 records; the Y axis was truncated at 150 for ease of viewing low record numbers elsewhere.

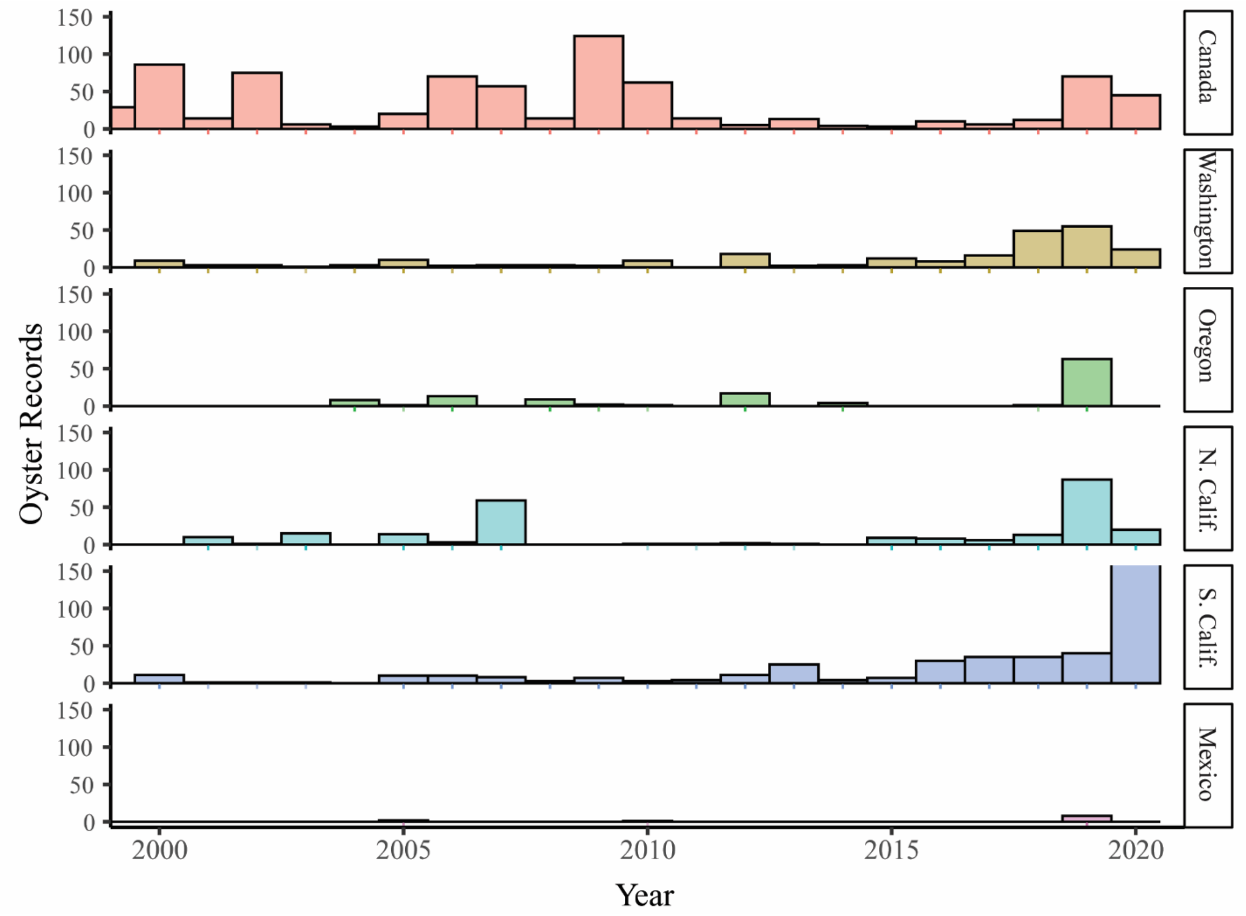

Figure C. Regional variation in larval network size and isolation.
Comparison among broad regions for both species for (A) larval network size, and (B) isolation. Each point represents larval network with at least three records used to calculate the index. North=Canada and Washington; Central=Oregon and California North; South=California South and Mexico.

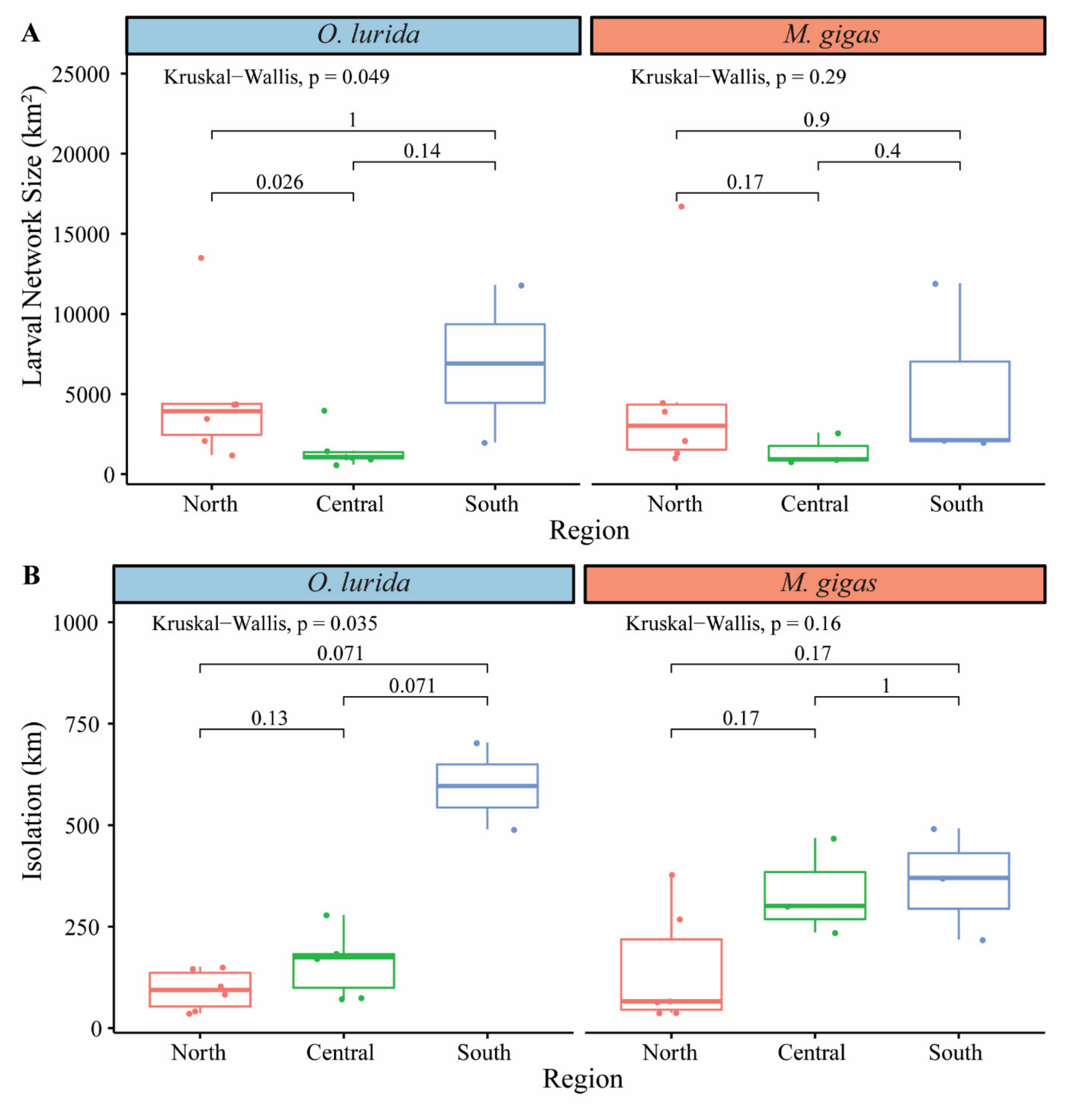

Figure D. Regional variation in distribution and abundance indices.
Comparison among states/provinces arranged from north to south for both species for (A) distribution index and (B) abundance index. Each point represents an estuary that had at least three records from which the index could be calculated. Kruskal-Wallis tests of differences among means within each plot and significance of H shown by p value.

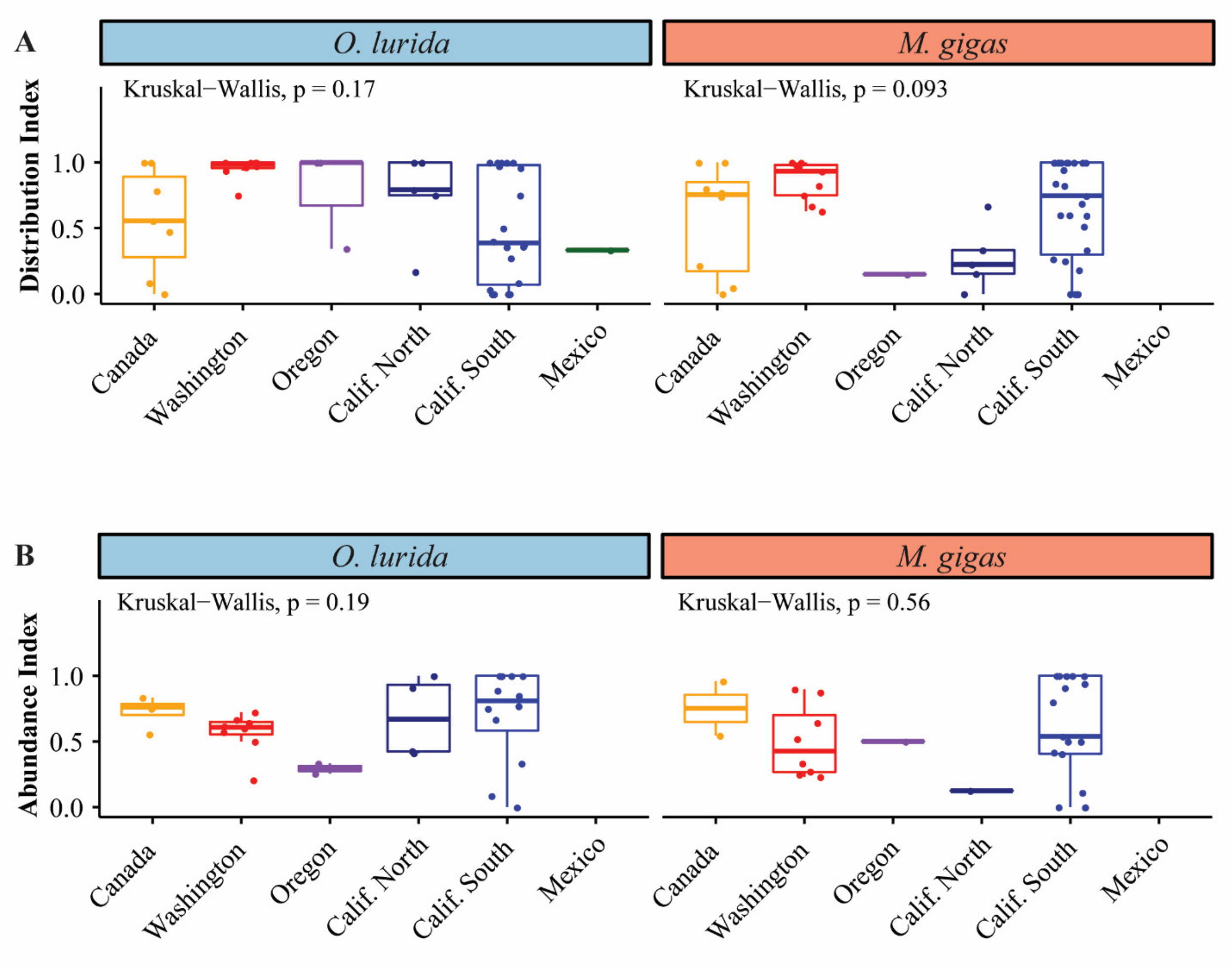

Figure E. Proportion of substrate types used by the two oysters.
All suitable records for coast combined across regions.

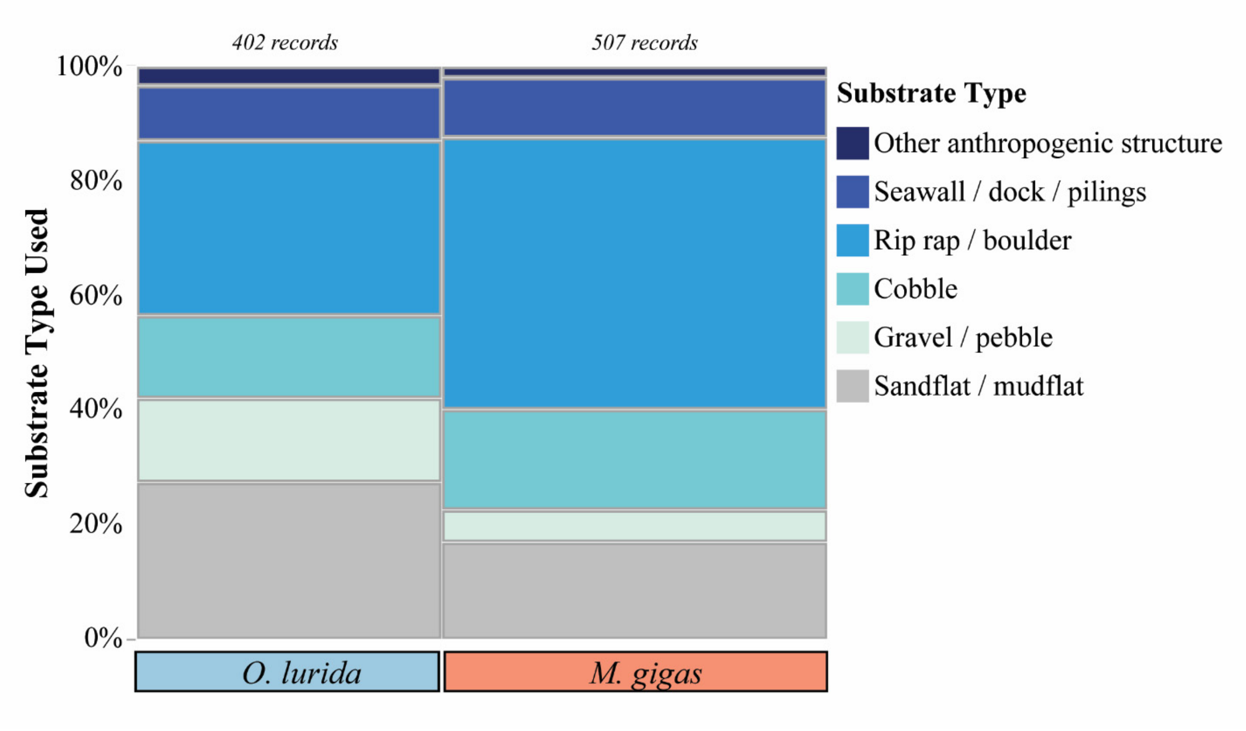

Figure F. Substrate availability vs. use.
For (A) San Diego Bay, Southern California and (B) Puget Sound, proportion of available substrate types is compared to what was actually documented to be used by *O. lurida* and *M. gigas*. Number of records of oyster substrate use shown in parentheses above each column for each species and estuary. Substrates with <10 records were not included.

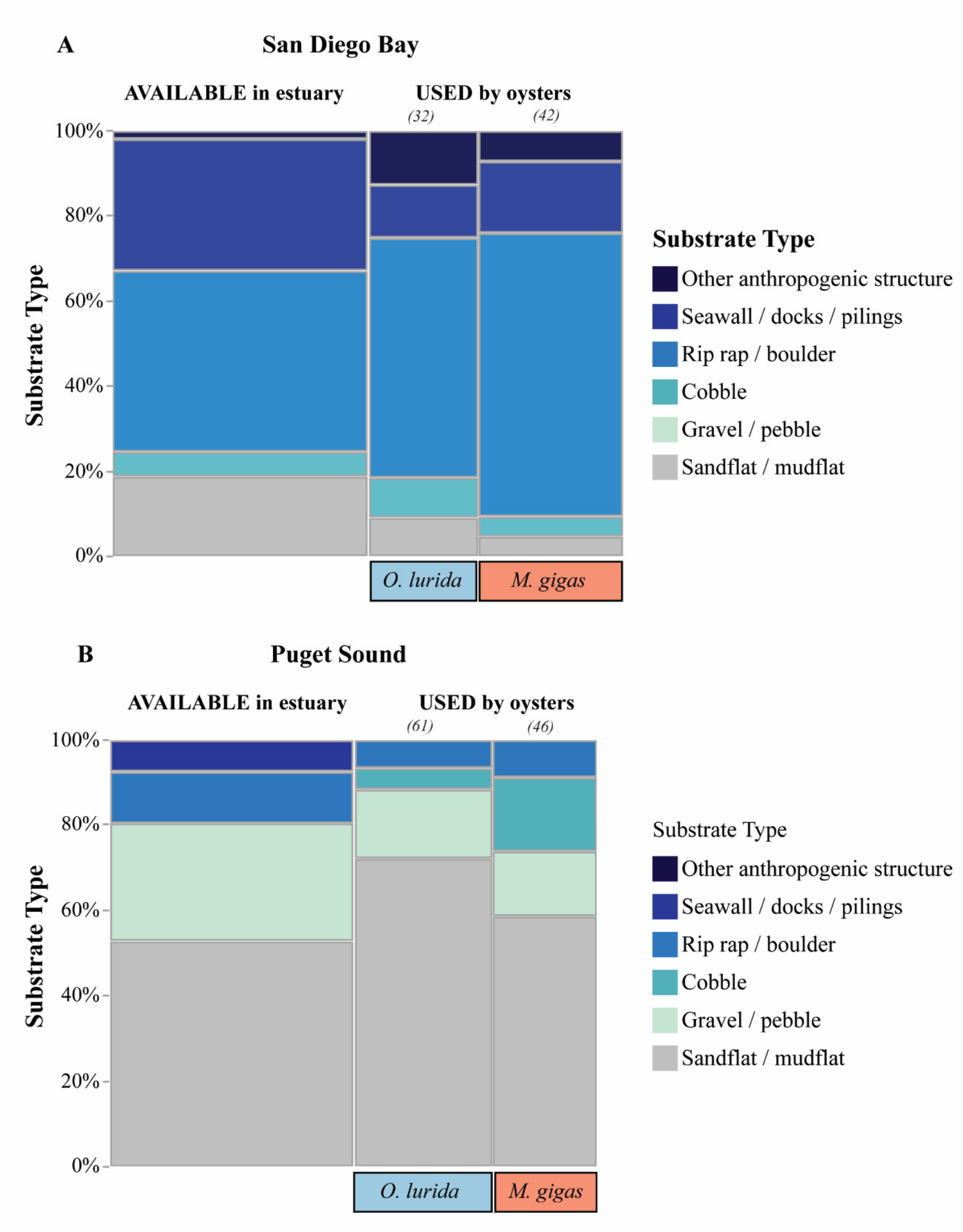

Figure G. Comparison of population structure and larval networks.
Phylogenetic tree of *O. lurida* modified from Silliman 2019 (Evol. Appl. 12:923–39) is shown for 19 estuaries from British Columbia to southern California using TreeMix to estimate relatedness based on one SNP per neutral locus. The tree is rooted in the southernmost populations and was color-coded by Silliman according to inferred phylogeographic region. To the right of the estuary names, we show the larval networks generated by our analysis, indicated by black 0-X labels. In almost all cases, separate branches on Silliman's phylogenetic tree correspond to larval networks we identified as separate based on the spatial analyses. The San Francisco Bay area is the only region where there are differences: Tomales Bay emerged as distinct from San Francisco Bay in Silliman's analysis while the two estuaries were part of a joint larval network in our analysis; conversely the Humboldt Bay population clustered with San Francisco Bay in Silliman's analysis but separate in ours.

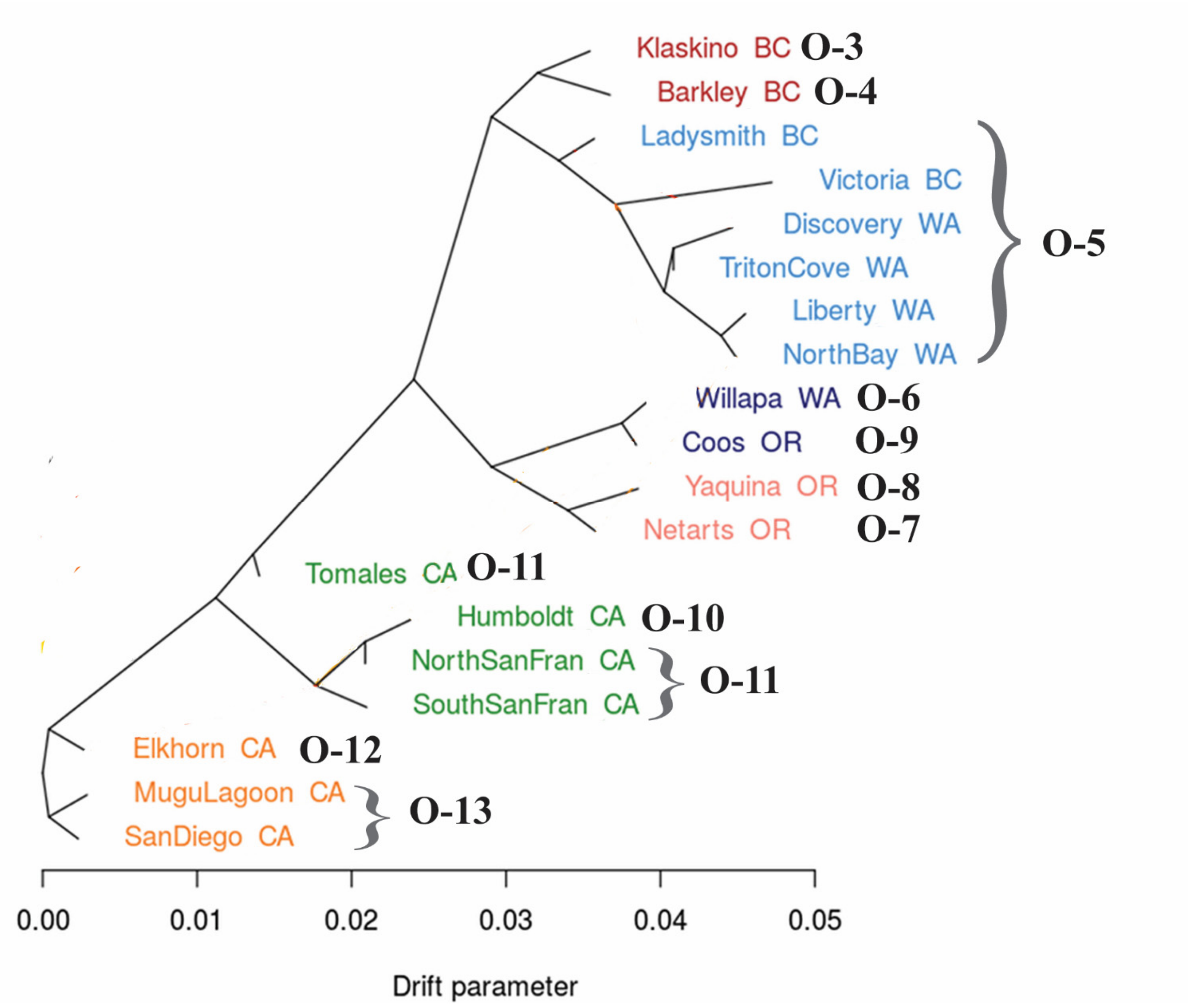

Supplement: S1 File — (PDF) [file pone.0263998.s001.pdf]
