# Supplementary material for: Mapping oysters on the Pacific coast of North America: A coast-wide collaboration to inform enhanced conservation
Source: PLoS One. 2022 Mar 17;17(3):e0263998. doi: 10.1371/journal.pone.0263998 (PMC8929589; doi:10.1371/journal.pone.0263998)

# THE OLYMPIA & PACIFIC OYSTER DATA PORTAL - INSTRUCTION MANUAL
# Mapping Oyster Distributions from British Columbia, Canada to Baja California, Mexico

## BACKGROUND

This project, jointly led by the Native Olympia Oyster Collaborative (NOOC) and The Pew Charitable Trusts (Pew), will create the first detailed, range-wide maps of current and historic distributions of the Olympia oyster (*Ostrea lurida*) to inform conservation and restoration strategies, and of the feral Pacific oyster (*Magallana gigas*) along the pacific coast of North America.

The data that will build this map come from YOU – anyone who knows of oyster localities in this region, and is willing to spend 5 minutes or less per site entering records. Thank you for participating in this ambitious cloud- and crowd-sourcing project!

There are two mechanisms for entering site data. One is to **fill out a spreadsheet with GPS coordinates** (you can read coordinates off Google Earth or use your phone). This may be the easiest way to enter data if you have a lot of sites where the same information will be repeated (same year of survey, same observer, etc.).

**QUICK MAP PORTAL USER'S GUIDE:**

1. Sign up for a public ArcGIS Online account
2. When signed in to AGOL, navigate to the Portal
3. Zoom to where you want to create a new record
4. Click "Edit" on the left top menu
5. Click "Create new oyster point location"
6. Drop your point on the map
7. Fill out the required fields marked by "*", and any other fields you want
8. Close the tab, and you're done!

The other mechanism for entering site data is to use our custom-built **interactive ArcGIS Online map, the Olympia & Pacific Oyster Data Portal (the Portal)**. **We recommend this for most users** and all the instructions below are for using this tool.

**This instructional guide will cover:**

- *Viewing the Portal and its data*
- *Adding oyster location point data*
- *Editing oyster location point data*

We've written a lot of instructions, but don't let that worry you. **The actual process is super easy** – you click to add a point, select some info from drop-down menus, type in your name and year, and you're done. It takes <5 minutes per site once you're familiar with it.

***Please note that this instruction manual refers to a version of the Portal that is currently closed to the addition of new data or edits to existing data.***

## Step 1: GET AN ESRI ArcGIS ONLINE ACCOUNT

If you don't yet have one, you'll need to **sign up for a free ArcGIS online (AGOL) account** here. It is necessary to have an account to add/edit data, but also important for tracking purposes (i.e., so that we know who entered what data).

## Step 2: VIEWING THE PORTAL AND ITS OYSTER DATA

When signed in to your AGOL account, **navigate to the Portal**. We recommend opening it in full screen format.

The map opens by default to the LEGEND view. You will be adding to and/or editing the layer titled "O. lurida & M. gigas points" which is turned on by default and appears as a grey circle: ◯

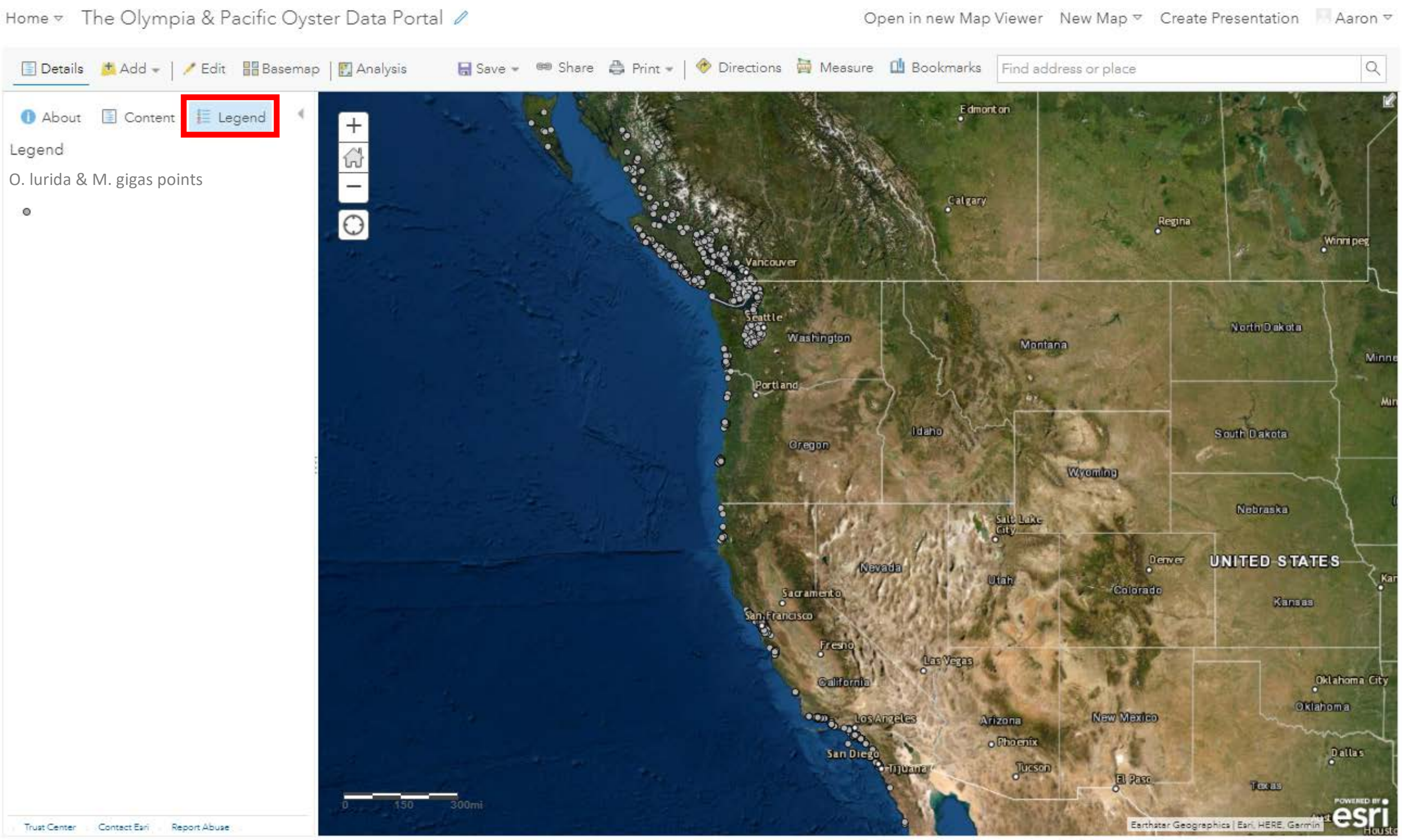

To familiarize yourself with the Portal and data, click an existing point. At right is a test example:

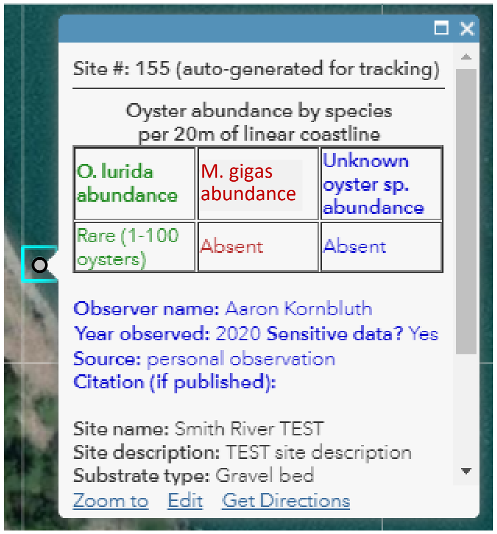


You'll notice several attribute fields for each point in the pop-up window:

- Auto-generated site number (*for tracking purposes only; cannot be edited*)
- Oyster abundance of: 1) *O. lurida*, 2) *M. gigas*, and 3) Unknown oyster species
- Observer name
- Year observed
- Sensitive data
- Site name
- Site description
- Substrate type
- General notes

There may also be an attachment(s), such as photos, videos, or documents. Each site, when entered, is automatically assigned a site number, listed at the top of the pop-up. This is for tracking purposes only.

Now **check out the CONTENT tab** where there are relevant layers pre-loaded:

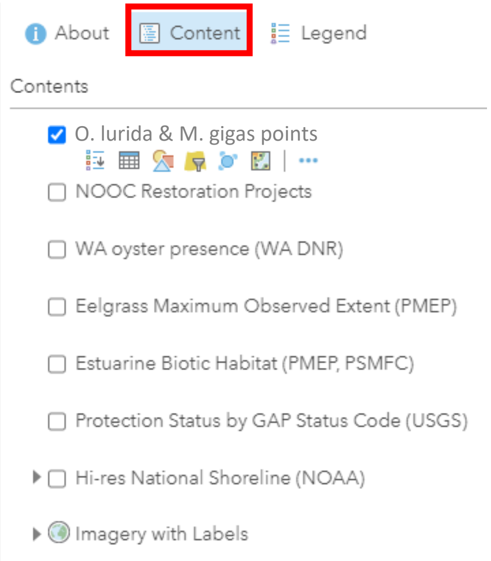


The editable layer – O. lurida & M. gigas points – will be turned on by default.

**You can turn layers on or off** at any point by clicking the radio button ☐ to the left of the layer title. These additional layers are intended to help you better locate your point data and provide additional detail that you may want to capture in the site description and/or notes field. A full description of each additional layer will be provided in a separate document.

You'll notice that some layers are not visible when you first load the Portal. You'll need to **zoom in to make some layers appear**.

You can also select a different basemap by selecting BASEMAP in the top menu. We recommend, however, using the default "Imagery with labels" base layer, as it provides the highest resolution and most detail to help you select the locations for your data points.

## Step 3: <u>ADDING</u> OYSTER LOCATION POINT DATA

Now you're ready to start adding data. You will be able to *view* points entered by other participants, but you will not be able to *edit* points created by others. Before you create a new oyster point location, you'll need to **zoom in to where you want to add the point**. You can use the mouse scroll wheel and pan the map in any direction, or you can **use the location search bar** at the top right of the window. You search by address, zip, or GPS coordinates.

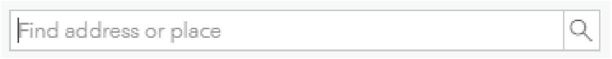


**Zoom in to the maximum extent** to create a new point with as much precision as possible. You may also want to turn on some of the additional layers under the CONTENT window to help you get your bearings.

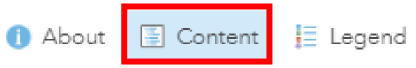


In places where it's difficult to see the shoreline, **we recommend turning on the layer "Hi-res National Shoreline" layer,** to reduce the likelihood of points being sited above mean high water (i.e., higher in elevation than oysters actually occur on the shoreline). In most places however, you can see the shoreline quite clearly from the default imagery.

To create your first oyster location point, **select EDIT from the top left menu**.

**Select "Create new oyster point location," and then drop the point on the map** where you know oysters are currently located or were historically located.

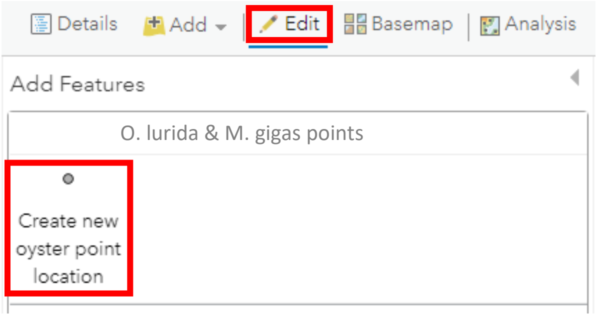

You'll get a pop-up window that look like this:

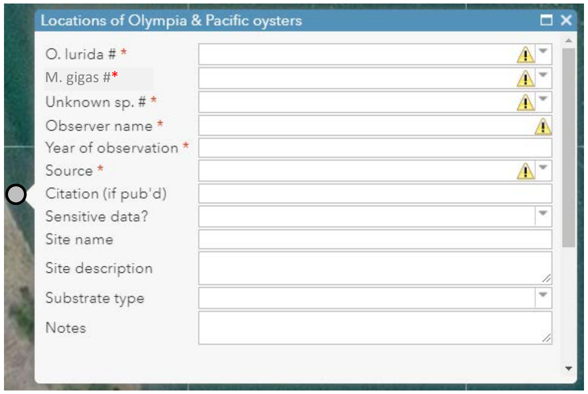


You have to **fill out the starred "*" (required) fields**. "Citation (if published)," "Sensitive data?," "site name," "site description," "substrate type," and "notes" are optional. Attachments (also optional) can be added by clicking "Choose File" at the bottom of the pop-up window.

*Oyster species abundance:*

You are required to **fill out abundance estimates for 3 oyster "species"**: the native *O. lurida*, feral populations of *M. gigas*, or "Unknown sp." We want information on both native and non-native species; even if one of them is absent, knowing that is helpful. The "Unknown" option is for people who do not know how to distinguish oysters in the field, or for iNaturalist records. Knowing there's some sort of oyster present is helpful even if we don't know which one.

We've simplified abundance options into 5 "bins"

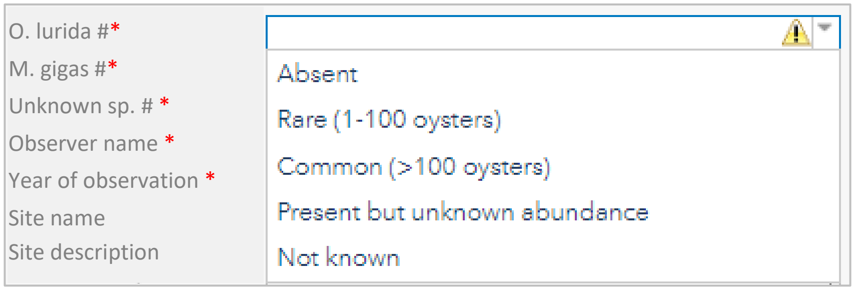


For each of three "species," you need to **select one of these five options**. Note that the numeric thresholds for "Rare" and "Common" are meant to apply to a 20-meter stretch of shoreline encompassing the point you entered for the site, i.e., 10m of intertidal on either side of the site. The size of this imaginary rectangle varies depending on how wide the intertidal is: in areas with gentle slopes, it might be 10x50m; in areas with steep slopes, it might be 10x5m. These abundance numbers are not densities and you don't need to worry too much about precision. You're really just trying to figure out of there are lots of oyster at this site or just a few. So if you have a sense of abundance even in the absence of quantitative data, we'd prefer the guess of Rare vs. Common rather than "Present." However, if you really have absolutely no idea whether they are Rare or Common, but know that they are there, then pick "Present but unknown abundance."

For rare cases where you enter subtidal data, consider the Rare/Common assessment to apply to a circle with 10m radius around your point.

If you know which oyster species occur at the site, enter "Absent" for the "Unknown Sp. #" – i.e., *there were zero unidentified oysters there*.

**For sites with no oysters:** points that identify a *total absence* of oysters should only be added in instances where they were actively looked for by people who would have noticed them (e.g., not absence from general species lists for broad surveys). **Dead shells should be scored as "absent**," but please include a comment in the Notes field. And if you know of an earlier record for that site, add a new point as "Rare," "Common," or "Present."

*Observer name*

**Enter your name, or if you're entering someone else's record, their name**. If you're entering a record on behalf of a team or organization, that's fine, but please clarify in the Notes field. If entering a record that has been previously published, please **provide the citation in the Citation drop-down field**. If entering lots of such records, please email XXXX to make sure those records are not already being entered by someone else, and to request use of the spreadsheet entry form, which may be easier because you can copy/paste that way.

*Year of observation*

**Enter the year of the observation** (not the year you are entering the record). Quick note, if the Portal adds a thousands separator comma to your year of observation (as in "2,020" instead of "2020"), don't worry about it.

If you have multiple years of monitoring data for the same exact site, and the abundance level was the same, please **enter the most recent year in the year field**, and then describe the earlier records in the Notes field (e.g., "same abundance of both species in 2000, 2005, and 2010"). If the abundance levels were different, feel free to create separate records for earlier years. (This database is not intended to be a repository for all details of interannual variation in monitoring data, but rather as a tool for knowing where they are now and how that has changed).

*Optional fields:* source, citation (if published), sensitive data, site name, site description, substrate type, and notes are optional, but desirable.

> *Source and citation*
> **Select the type of data.** Choose the descriptor that best fits (you can use your own discretion in deciding between "personal observation" and "monitoring data," etc.). Most importantly, if this is a published record (including theses, publicly available reports, etc.) please enter the full citation in the Citation field. NOOC is attempting to collect all publications related to oysters on this coast; if your citation is obscure (a local agency report, an undergraduate senior thesis, etc.) please email us PDF of the source.
>
> *Sensitive data*
> **Click "yes" if you want the exact location of this point obscured** in any future maps of the estuary or interactive mapping websites. The exact coordinates will be archived, but in public versions they will be "fuzzy," showing a larger area around the point, or will be omitted entirely (feel free to provide more detail on sensitivity in Notes).
>
> *Substrate type*
> **Select the single most common substrate that oysters are found on** at the site (if they are present), excluding the use of other oysters as substrate (i.e., what is the oyster individual or cluster found on?)

*Saving your data entries:* Once you've filled out the pop-up window to your satisfaction, **save the new point by clicking on the map outside the pop-up window. Note that <u>it will not save</u> if you haven't filled out the required fields (*).** To create more points, follow the steps above.

## Step 4: EDITING OYSTER LOCATION POINT DATA

You can revisit your records to make edits/improvements to locations and the attribute data. You will be able to *view* the points entered by other participants, but you will not be able to *edit* them. **To edit your own data, select "Edit" in the top left menu** (see Step 3 above)**.**

To edit existing point data, *don't select* "Create new oyster point location," just **select the point on the map** that you wish to edit, and click "Edit" at the bottom of the pop-up window. You can drag the point to a new place if you would like to change its location. You can also edit the attribute information in the pop-up window.

To save your updates, select "CLOSE" in the pop-up window. To delete a point, select "DELETE" in the pop-up window. No one but you can delete your points.

You may find it useful to use the MEASURE tool at the top right menu. This allows you to calculate areas, lengths, or locations (i.e., GPS coordinates). Use this, for example, if you know the distance from shore of a particular *O. lurida* cluster (see blue line measurement example in the image at right).

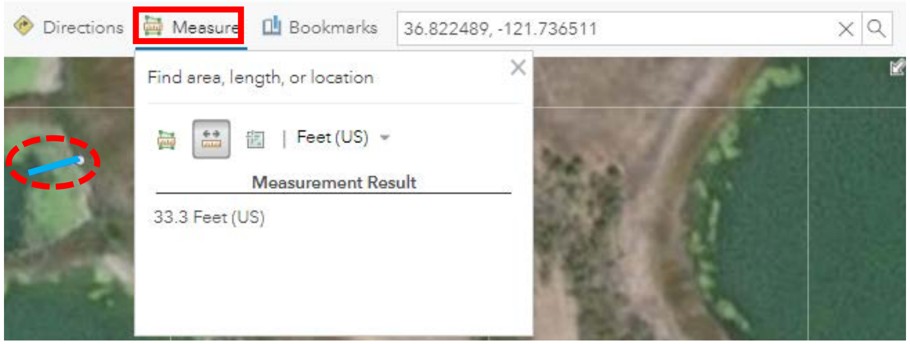

**Editing using the table**

You may want to edit multiple, previously created points quickly. You can do that using the attribute table that “lives behind the walls” of the oyster layer. To do that, select 1) Details, 2) Content, and then 3) click the table icon.

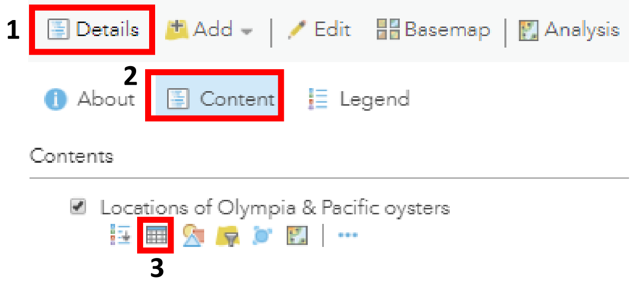


You’ll only be able to edit the points that you have created. To do so, **double-click on the cell you want to edit**.

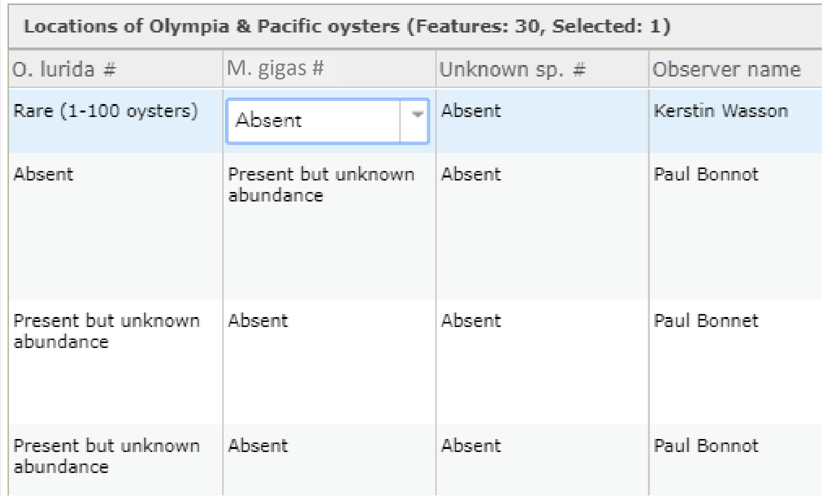

Locations of Olympia & Pacific oysters (Features: 30, Selected: 1)

| O. lurida # | M. gigas # | Unknown sp. # | Observer name |
|---|---|---|---|
| Rare (1-100 oysters) | Absent | Absent | Kerstin Wasson |
| Absent | Present but unknown abundance | Absent | Paul Bonnot |
| Present but unknown abundance | Absent | Absent | Paul Bonnet |
| Present but unknown abundance | Absent | Absent | Paul Bonnot |

## STEP 5 (OPTIONAL): CREATING OVERLAPPING POINTS

You may need to add two or more points to the same location to identify a current (2000-2020) observation and one or more historic (before 2000) observations. To do this, create your first point following Step 3 above. **Make sure you’re zoomed in to the maximum extent,** then create a second (or third, or fourth …) point in the same location as the first, and fill out the required and optional fields.

To flip among multiple points, click on the point that you can see (it’ll be the most recent one you created), and then click the white arrow at the top right of the pop-up window to navigate among multiple points. If you need to edit one of them, select edit at the bottom of the pop-up window.

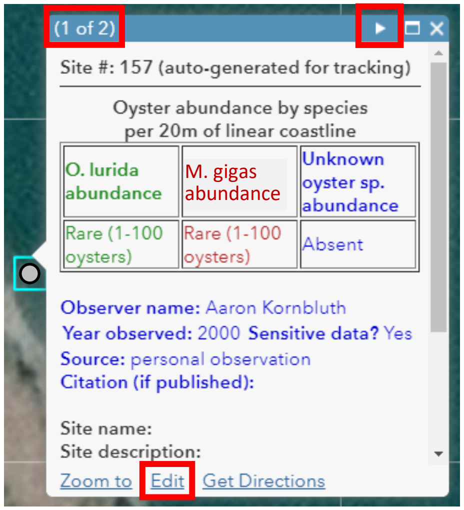

Supplement: S2 File — (PDF) [file pone.0263998.s002.pdf]
