# Supplementary material for: Mapping oysters on the Pacific coast of North America: A coast-wide collaboration to inform enhanced conservation
Source: PLoS One. 2022 Mar 17;17(3):e0263998. doi: 10.1371/journal.pone.0263998 (PMC8929589; doi:10.1371/journal.pone.0263998)
Supplement: S4 File — (DOCX) [file pone.0263998.s004.docx]

**S4 File. Interactive ArcGIS on-line map - the “The Olympia & Pacific Oyster Data Portal” - on the NOAA GeoPlatform.** <https://noaa.maps.arcgis.com/apps/View/index.html?appid=a49a0bc8a0764fc799d81f6652b7a13b> . All geospatial metadata for the Portal is available on the NOAA InPort NMFS Enterprise Data Management Program site at <https://www.fisheries.noaa.gov/inport/item/65431> .
